# iMLGAM: Integrated Machine Learning and Genetic Algorithm‐driven Multiomics analysis for pan‐cancer immunotherapy response prediction

**DOI:** 10.1002/imt2.70011

**Published:** 2025-03-08

**Authors:** Bicheng Ye, Jun Fan, Lei Xue, Yu Zhuang, Peng Luo, Aimin Jiang, Jiaheng Xie, Qifan Li, Xiaoqing Liang, Jiaxiong Tan, Songyun Zhao, Wenhang Zhou, Chuanli Ren, Haoran Lin, Pengpeng Zhang

**Affiliations:** ^1^ Liver Disease Center of Integrated Traditional Chinese and Western Medicine, Department of Radiology, Zhongda Hospital, Medical School Southeast University, Nurturing Center of Jiangsu Province for State Laboratory of AI Imaging & Interventional Radiology (Southeast University) Nanjing China; ^2^ Department of Thoracic Surgery The First Affiliated Hospital of Nanjing Medical University Nanjing China; ^3^ Department of Thoracic Surgery, Nanjing Chest Hospital Nanjing China; ^4^ Afliated Nanjing Brain Hospital Nanjing Medical University Nanjing China; ^5^ Department of Oncology, Zhujiang Hospital Southern Medical University Guangzhou China; ^6^ Department of Urology, Changhai Hospital Naval Medical University (Second Military Medical University) Shanghai China; ^7^ Department of Plastic Surgery, Xiangya Hospital Central South University Changsha China; ^8^ Department of Thoracic Surgery The First Affiliated Hospital of Soochow University Suzhou China; ^9^ Chongqing Key Laboratory of Molecular Oncology and Epigenetics The First Affiliated Hospital of Chongqing Medical University Chongqing China; ^10^ Department of Lung Cancer, Tianjin Lung Cancer Center, National Clinical Research Center for Cancer, Key Laboratory of Cancer Prevention and Therapy, Tianjin's Clinical Research Center for Cancer Tianjin Medical University Cancer Institute and Hospital Tianjin China; ^11^ Department of Plastic Surgery The First Affiliated Hospital of Wenzhou Medical University Wenzhou China; ^12^ Department of Oncology The Affiliated Huai'an Hospital of Xuzhou Medical University, the Second People's Hospital of Huai'an Huai'an China; ^13^ Department of Laboratory Medicine Northern Jiangsu People's Hospital Affiliated to Yangzhou University Yangzhou China

**Keywords:** gene‐pair, genetic algorithms, immune checkpoint blockade, immunotherapy, pan‐cancer

## Abstract

To address the substantial variability in immune checkpoint blockade (ICB) therapy effectiveness, we developed an innovative R package called integrated Machine Learning and Genetic Algorithm‐driven Multiomics analysis (iMLGAM), which establishes a comprehensive scoring system for predicting treatment outcomes through advanced multi‐omics data integration. Our research demonstrates that iMLGAM scores exhibit superior predictive performance across independent cohorts, with lower scores correlating significantly with enhanced therapeutic responses and outperforming existing clinical biomarkers. Detailed analysis revealed that tumors with low iMLGAM scores display distinctive immune microenvironment characteristics, including increased immune cell infiltration and amplified antitumor immune responses. Critically, through clustered regularly interspaced short palindromic repeats screening, we identified Centrosomal Protein 55 (*CEP55*) as a key molecule modulating tumor immune evasion, mechanistically confirming its role in regulating T cell‐mediated antitumor immune responses. These findings not only validate iMLGAM as a powerful prognostic tool but also propose *CEP55* as a promising therapeutic target, offering novel strategies to enhance ICB treatment efficacy. The iMLGAM package is freely available on GitHub (https://github.com/Yelab1994/iMLGAM), providing researchers with an innovative approach to personalized cancer immunotherapy prediction.

## Highlights

- Developed integrated Machine Learning and Genetic Algorithm‐driven Multiomics analysis (iMLGAM), an integrated machine learning and genetic algorithm‐driven multiomics analysis package for immunotherapy response prediction.
- iMLGAM scoring system demonstrates superior performance in predicting immune checkpoint blockade (ICB) treatment outcomes across multiple cohorts.
- Low iMLGAM scores associate with enhanced immune cell infiltration and amplified antitumor responses.
- *CEP55* identified as a crucial regulator of T cell‐mediated antitumor immunity and ICB efficacy.

## Graphical abstract

We developed an integrated Machine Learning and Genetic Algorithm‐driven Multiomics analysis (iMLGAM), an R package that combines various machine learning algorithms with genetic algorithms and multi‐omics data to predict responses to immune checkpoint blockade (ICB) therapy. Utilizing pan‐cancer tumor data, we established the iMLGAM scoring system to forecast ICB therapy outcomes. The system was validated through experimental methods and implemented as a Shiny web application. Clinical cohort validation further demonstrated its reliability in optimizing immunotherapy treatment decisions.

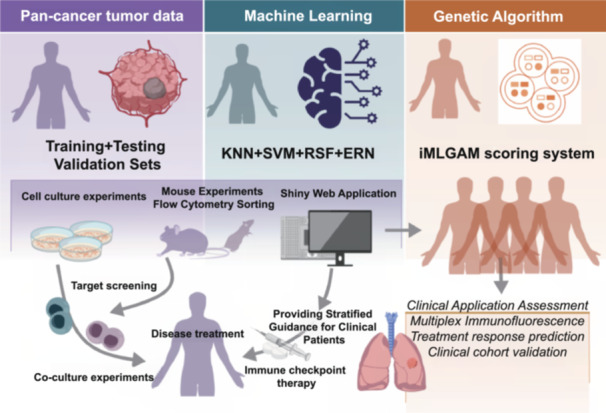

## INTRODUCTION

The field of cancer therapeutics was fundamentally transformed through the introduction of immune checkpoint blockade (ICB), which yielded remarkable improvements in patient survival outcomes [1]. Although ICB has demonstrated encouraging therapeutic effects in certain tumor types (such as melanoma [2], non‐small cell lung cancer (NSCLC) [3], and renal cell carcinoma (RCC) [4]), immunotherapy is not a universal solution for most cancer patients. Research indicates significant variations in immunotherapeutic responses across different cancer types, with many patients failing to achieve notable clinical benefits. For instance, patients with colorectal cancer, pancreatic cancer, and prostate cancer exhibit relatively poor responses to ICB, highlighting the importance of personalized treatment. The use of ICB among eligible cancer patients in the United States has increased dramatically, rising from 1.54% in 2011 to 43.63% by 2018 [5]. Despite widespread adoption, clinical efficacy remained limited, with favorable responses being observed in merely 12.46% of treated subjects [5]. Significant variations in therapeutic outcomes were documented across different malignancies and individual cases [6]. Scientific investigations identified multiple inflammation‐associated parameters as potential response predictors for ICB therapy, encompassing tumor‐infiltrating lymphocytes (TILs), tumor mutation burden (TMB), Immunoscore, and gastrointestinal oncological microbiome patterns. However, these biological markers demonstrated restricted value [7]. The field continues to lack a universally applicable predictive marker for accurate ICB response forecasting across various cancer types, which has hindered both optimal patient selection and the advancement of combination therapeutic strategies.

The clinical outcomes and therapeutic response to ICB were substantially influenced by the tumor immune microenvironment (TIME), which comprised diverse immunological cellular components [8, 9]. Elucidating the associations between TIME characteristics and ICB therapeutic efficacy was considered crucial for enhancing patient stratification and optimizing immunotherapeutic interventions. However, the complex interactions among immune cells can result in conflicting interpretations, highlighting the need for more advanced analytical methods. Traditional analytical techniques, including immunohistochemistry (IHC) and flow cytometry, which were routinely employed for TIME examination, exhibited limitations in the concurrent evaluation of multiple cellular markers. While single‐cell RNA sequencing (scRNA‐seq) provided detailed transcriptomic analysis at high resolution, its clinical implementation was hampered by economic considerations and rigorous sample requirements [10, 11]. By comparison, bulk RNA sequencing (bulk RNA‐seq) exhibited superior adaptability with suboptimal specimens and generated comprehensive transcriptional profiles from heterogeneous tissue samples. Consequently, the investigation of immune‐associated gene expression through bulk RNA‐seq emerged as a dependable approach for TIME characterization [12].

In the field of transcriptomic data analysis, ensemble learning methodologies have been demonstrated to provide enhanced predictive accuracy for outcomes when compared to individual machine learning approaches [13, 14, 15]. However, several technical challenges have been identified, including the risk of overfitting, handling of the heterogeneity of sequencing platforms, and the lack of automated protocols for the identification of basic learners [16]. To overcome these limitations, we developed integrated Machine Learning and Genetic Algorithm‐driven Multiomics analysis (iMLGAM), an R package and its novel scoring system for predicting outcomes in cancer patients undergoing ICB therapy. This R package integrates several key components: a gene‐pairing protocol was implemented to reduce batch effects [17], the adaptive best subset selection (ABESS) algorithm was utilized for feature optimization, and a genetic algorithm (GA) was employed for automated basic learner optimization [18]. These developments culminated in the creation of a Shiny‐based online platform (https://ici-theaphy-gms.shinyapps.io/my_shiny_app/) for iMLGAM score computation, providing a prognostic evaluation for diverse cancer patients undergoing ICB treatment. This innovative methodology was designed to establish a more robust and adaptable response prediction system, with potential implications for therapeutic outcome enhancement and clinical strategy formulation in cancer immunotherapy.

## RESULTS

### Development of iMLGAM score

**Figure 1:**
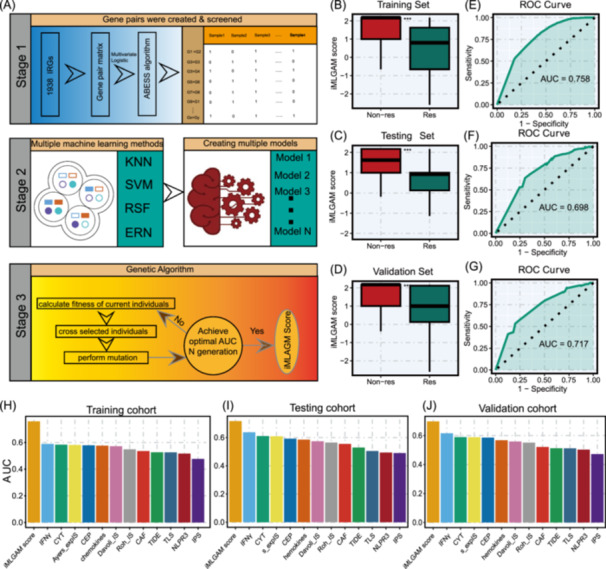
Development and validation of integrated Machine Learning and Genetic Algorithm‐driven Multiomics analysis (iMLGAM) score for predicting immunotherapy response. (A) Comprehensive flow chart detailing the systematic process of training, testing, and validation of the iMLGAM score, highlighting the integration of genetic algorithms (GAs) and ensemble learning methods for multi‐omics data analysis. (B–D) Comparative distribution of iMLGAM scores demonstrating clear differentiation between immunotherapy responders and nonresponders across training, testing, and independent validation cohorts, revealing the score's potential for treatment response stratification. (E–G) Receiver operating characteristic (ROC) curves illustrating the predictive accuracy of iMLGAM scores in identifying potential immunotherapy benefits, showcasing the method's robust performance across different patient cohorts. (H–J) Comparative performance analysis comparing iMLGAM scores against existing published molecular signatures, evaluated through area under the curve (AUC) metrics, demonstrating the novel approach's superior predictive capabilities in multiple independent datasets.

To develop the iMLGAM scoring system, we implemented a comprehensive machine learning workflow integrating multiple algorithmic approaches. The construction process of the iMLGAM score was performed entirely within the training cohort. Feature processing was initially performed on the training set to minimize batch effects, yielding 187,953 immune‐related gene pairs (IRGPs). Through multivariable logistic regression with cancer type adjustment (*p* < 0.001) and receiver operating characteristic (ROC) curve analysis (area under the curve (AUC) > 0.6), we identified 2537 key IRGPs in the training cohort (Table S1). We identified five IRGPs from 2537 key IRGPs using the ABESS algorithm, specifically the gene pairs *BMP2* and *SELE*, *CD274* and *SH3TC1*, *CHST15* and *LAG3*, *CKLF* and *TLR7*, and *ESCO2* and *RXRA*, which were used as basic features to construct the iMLGAM score. To enhance predictive performance, we employed four distinct machine learning regression algorithms: Elastic Net (Enet), Random Forest (RF), Support Vector Machine (SVM), and K‐Nearest Neighbors (KNN). Parameter optimization was conducted using 10‐fold cross‐validation (CV) and grid search, generating 50 basic learners (m1–m50) for subsequent analysis (Table S2). These learners were integrated using a stacking strategy to create an ensemble model, designated as the iMLGAM score. The model's robustness was fundamentally dependent on both the diversity and accuracy of its constituent components. To streamline the traditionally manual optimization process, we implemented a GA to enhance the ensemble model's accuracy. The optimized iMLGAM score incorporated ten carefully selected models: m3, m5, m6, m16, m19, m23, m24, m29, m40, and m46. This refined ensemble demonstrated remarkable algorithmic diversity, comprising three Enet models (m3, m5, m6), two KNN models (m40, m46), two RF models (m16, m19), and three SVM models (m23, m24, m29), validating the GA's effectiveness in model selection. The final iMLGAM score was constructed using stepwise logistic regression as the ensemble learning method to combine the predicted values from these optimized learners in the training set (Figure 1A).

### Assessment of iMLGAM score

To evaluate the iMLGAM score's efficacy in predicting clinical responses, we applied it to the training, validation, and test cohorts. The score effectively distinguished between response and nonresponse groups in all three cohorts, with lower scores indicating a greater likelihood of benefiting from ICB therapy (all *p* < 0.001) (Figure 1B–D). ROC curve analysis revealed substantial AUC values: 0.76 for the training set (Figure 1E), 0.70 for the testing set (Figure 1F), and 0.72 for the validation set (Figure 1G). Based on ROC curve analysis of the training cohort, we obtained the optimal threshold and stratified patients into high and low iMLGAM score groups. In the training set, the metrics are as follows: F1 score 0.78, true positive rate 0.71, true negative rate 0.67, false positive rate 0.33, and false negative rate 0.29. For the testing set, the metrics are: F1 score 0.74, true positive rate 0.64, true negative rate 0.70, false positive rate 0.30, and false negative rate 0.36. In the validation set, we observed: F1 score 0.68, true positive rate 0.57, true negative rate 0.76, false positive rate 0.24, and false negative rate 0.43. The high iMLGAM score group demonstrated significantly poorer overall survival (OS) across all cohorts (*p* < 0.05) (Figure S1A–C). Time‐dependent ROC curves further validated the iMLGAM score's effectiveness in predicting OS for patients receiving ICB therapy (Figure S1D–F). We also assessed the iMLGAM score's performance across various tumor types. In RCC, urothelial carcinoma (UC), melanoma, and gastric cancer (GC), the score was consistently lower in the response group (*p* < 0.05), with ROC‐AUC values exceeding 0.6 (Figure S2A–J). No significant difference was found in glioblastoma multiforme (GBM) (Figure S2K,L), likely due to the limited sample size for this tumor type. These results highlight the iMLGAM score's strong predictive capability across multiple cancers, emphasizing its potential as a valuable tool for guiding ICB therapy decisions.

### Comparison of iMLGAM score with other features and webtool development

**Figure 2:**
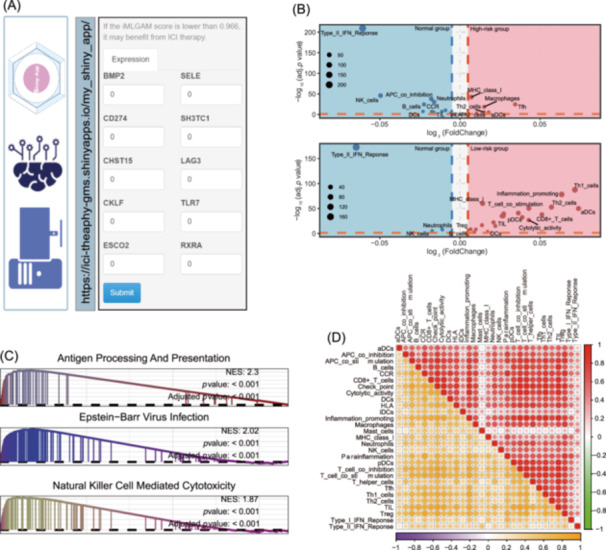
iMLGAM score associates with distinct immune signatures and molecular pathways. (A) The page of iMLGAM score web‐tool. (B) Volcano plot analysis depicting differential enrichment of 29 immune signatures between high and low iMLGAM score groups. Red points indicate signatures enriched in tumor tissue; blue points represent signatures enriched in normal tissue. (C) Gene set enrichment analysis (GSEA) comparing molecular pathways between high and low iMLGAM score groups. (D) Correlation matrix visualization of 29 immune signatures, comparing low iMLGAM score group (upper right panel) versus high iMLGAM score group (lower left panel). iMLGAM, integrated Machine Learning and Genetic Algorithm‐driven Multiomics analysis.

Recent advancements in sequencing technologies and machine learning have facilitated the creation of predictive signatures for immunotherapy response. Various signatures, derived from distinct biological characteristics and molecular types, have demonstrated predictive value for ICB therapy. We systematically evaluated 12 published signatures alongside the iMLGAM score, finding that the iMLGAM score consistently outperformed others in predictive performance across training, testing, and validation cohorts (Figure 1H–J). Using comprehensive clinical data from the Mariathasan, Braun, OAK, and Riaz cohorts, we performed ROC curve analyses to compare the iMLGAM score's performance against PD‐L1 expression and TMB. The iMLGAM score consistently exceeded both PD‐L1 and TMB in these cohorts (Figure S3A–D). Multivariate logistic regression further established the iMLGAM score as an independent predictor of clinical response (all *p* < 0.05) (Figure S3E–H). To enhance accessibility for the research community, we developed a user‐friendly web tool for the iMLGAM score, shown in Figure 2A and available at https://ici-theaphy-gms.shinyapps.io/my_shiny_app/.

### Comprehensive immune landscape analysis

To further explore the relationship between the immune landscape and the iMLGAM score, we conducted a multiomics analysis of the TCGA cohort. Using the formula and cutoff from the training cohort, we stratified the TCGA cohort into high and low iMLGAM score groups (Figure S4A). Genomic analysis revealed significantly larger fractions of leukocytes, lymphocytes, and TILs in the low iMLGAM score group (*p* < 0.001) (Figure S4B–D). Deep learning estimates of TIL fractions from hematoxylin and eosin (H&E) stained slides corroborated these findings (*p* < 0.001) (Figure S4E). The low iMLGAM score group also had a higher proportion of immune‐stimulatory cells, particularly CD8^+^ T cells (*p* < 0.001) (Figure S4F). Further analysis using immune infiltration scores from Danaher et al. (Figure S4G) and immune signature scores (Figure S4H) confirmed a greater abundance of immune cells in the low iMLGAM score group (all *p* < 0.05). Unsupervised clustering based on immune signature scores revealed two distinct immune patterns: high and low immune infiltration, with the high infiltration group significantly enriched in cases from the low iMLGAM score group (*p* < 0.001) (Figure S4I,J). In the low iMLGAM score group, immune signature scores were significantly higher at tumor sites compared to normal sites, whereas the high iMLGAM score group showed lower scores at tumor sites than at normal sites (Figure 2B). The low iMLGAM score group exhibited stronger correlations among immune activities than the high iMLGAM score group (Figure 2D and Figure S5A). Gene set enrichment analysis (GSEA) identified 33 significantly enriched pathways in the low iMLGAM score group, including 19 immune‐related pathways such as “natural killer cell‐mediated cytotoxicity” (all *p* adjust < 0.05) (Figure 2C). No immune‐related pathway enrichment was observed in the high iMLGAM score group. Low iMLGAM score tumors were associated with significantly higher cytolytic activity scores (*p* < 0.001) (Figure S5B), while the high iMLGAM score group had a greater abundance of fibroblasts (*p* < 0.05) (Figure S5C). These findings indicate that the low iMLGAM score group is characterized by abundant immune cells at tumor sites, which may enhance responses to ICB therapy. In contrast, the prevalence of fibroblasts in the high iMLGAM score group could contribute to immune escape mechanisms. Additionally, the low iMLGAM score group exhibited higher chemokine expression (*p* < 0.05) (Figure S5D), suggesting that chemokine enrichment may drive the enhanced immune response observed in this group.

### Molecular and genomic determinants of tumor immunogenicity

We first examined key determinants of tumor immunogenicity between the low and high iMLGAM score groups. The low iMLGAM score group exhibited higher mutation rates and neoantigen burdens (*p* < 0.05), along with significantly greater T cell receptor (TCR) and B cell receptor (BCR) diversity (*p* < 0.001). Additionally, this group demonstrated increased CNV burden and aneuploidy (*p* < 0.001), while high iMLGAM score patients displayed lower intertumoral heterogeneity (ITH) (*p* < 0.001) (Figure S6A). These characteristics suggest a more immunogenic tumor microenvironment, typically associated with better responses to ICB therapy. The higher ITH may also provide a broader range of neoantigens, enhancing immune recognition and attack, indicating that low iMLGAM score patients may benefit more from immunotherapy. To further explore the mutational landscape, we analyzed somatic mutation data from the TCGA data set, identifying four distinct mutational signatures: SBS10b, SBS7a, SBS6, and SBS3 (Figure S6B). Notably, SBS10b, SBS3, and SBS6 had significantly higher frequencies in the low iMLGAM score group (*p* < 0.05) (Figure S6C). The SBS6 signature, linked to deficiencies in DNA mismatch repair, may influence immune responses [19]. We also calculated enrichment scores for oncogenes across 10 common oncogenic pathways. The low iMLGAM score group showed higher scores for the cell cycle and TP53 pathways, while the high iMLGAM score group had enriched pathways, including HIPPO, MYC, NOTCH, NRF2, RAS, TGF‐Beta, and WNT (*p* < 0.001) (Figure S6D). The WNT pathway is known to be involved in immune exclusion [20]. Furthermore, the high iMLGAM score group exhibited reduced expression of MHC I‐ and II‐related antigen‐presenting molecules compared to the low iMLGAM score group (*p* < 0.001), indicating intrinsic immune escape (Figure S6E). In contrast, the low iMLGAM score group showed increased expression of most MHC genes, reflecting enhanced immunogenicity. Additionally, immune checkpoint molecules (e.g., PD‐1, PD‐L1, and CTLA4) and costimulatory molecules were more highly expressed in the low iMLGAM score group (*p* < 0.001) (Figure S6E), suggesting their role in promoting responses to ICB therapy in this group.

### Copy number features associated with iMLGAM score groups

Significant disparities in chromosomal aberrations were observed between the high and low iMLGAM score groups (Figure S7A). The low iMLGAM score group exhibited focal amplifications of immune genes, including PD‐L1 and PD‐L2, located at 9p24.1, in contrast to the high iMLGAM score group (Figure S7B,C). Venn diagram analysis identified 319 shared genes in amplified chromosomal regions, with 217 genes unique to the high iMLGAM score group and 1225 unique to the low iMLGAM score group (Figure S7D). Gene Ontology annotation of these uniquely amplified genes revealed significant enrichment of immune‐related processes, particularly “T cell costimulation” and “B cell proliferation,” in the low iMLGAM score group (Figure S7E). Conversely, the high iMLGAM score group was enriched in “regulation of fibroblast proliferation” but showed no enrichment in immune‐related processes. This aligns with earlier findings of increased immune cell presence in the low iMLGAM score group and higher fibroblast levels in the high iMLGAM score group. Notably, PD‐L1 and PD‐L2, located within the amplification peak at 9p24.1 specific to the low iMLGAM score group, were linked to both immune‐related biological processes, indicating their potential role in modulating immune status (Figure S7F). mRNA expression analysis from the TCGA cohort confirmed significantly higher expression levels of PD‐L1 and PD‐L2 in the low iMLGAM score group (Figure S7G), supporting the CNV data. These findings suggest that tumor‐specific CNVs contribute to the differences in immune infiltration patterns.

### Validation of iMLGAM score's predictive value in an independent clinical cohort

**Figure 3:**
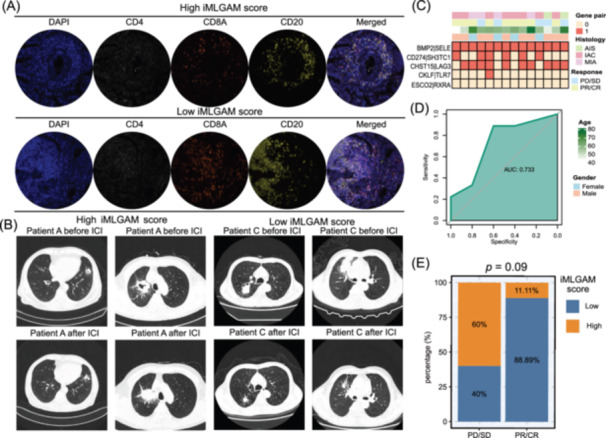
Clinical validation of iMLGAM score in the in‐house cohort. (A) Representative multiplex immunofluorescence images showing DAPI, CD4, CD8, and CD20 staining in high and low iMLGAM score groups. (B) Radiological assessment comparing pre‐ and post‐immunotherapy imaging in high and low iMLGAM score groups. (C) Baseline clinical characteristics of the in‐house cohort. (D) ROC curve analysis of iMLGAM score for predicting clinical response in the in‐house cohort. (E) iMLGAM score distribution between response and nonresponse groups.

We conducted multiplex immunofluorescence staining on NSCLC patient tissue samples using DAPI, CD4, CD8, and CD20 markers. Analysis revealed that specimens from the low iMLGAM score cohort had significantly higher infiltration of CD8^+^ T cells, and B lymphocytes compared to the high iMLGAM score group (Figure 3A, Figure S8A,B). Post‐immunotherapy imaging evaluated patient responses according to RECIST criteria (Figure 3B). The iMLGAM score's predictive value for neoadjuvant immunotherapy outcomes was confirmed through ROC analysis, yielding a strong AUC of 0.73 (Figure 3C,D). Although the responder group included a notable number of cases from the low iMLGAM score group (Figure 3E), this did not reach statistical significance (*p* = 0.09), likely due to the limited sample size (*n* = 14).

### Identification of therapeutic targets

**Figure 4:**
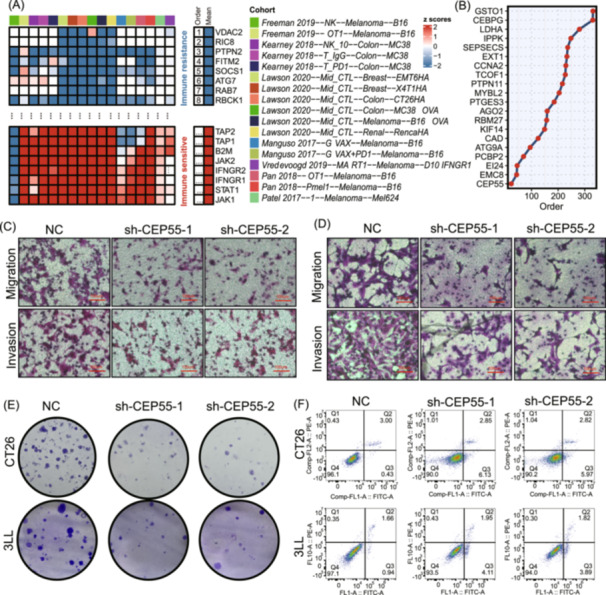
Clustered regularly interspaced short palindromic repeats (CRISPR) screening and functional analysis of centrosomal protein 55 (CEP55) based on iMLGAM score. (A) Gene ranking by antitumor immunity knockout effects from 17 CRISPR datasets. (B) Univariate Cox regression analysis of high iMLGAM score prognostic genes, with CEP55 as the top candidate from CRISPR screen data. Migration and invasion assessment of CT26 (C) and 3LL (D) cells after CEP55 knockdown using Transwell assays. (E) Colony formation assays showing inhibition of cancer cell proliferation following CEP55 knockdown. (F) Flow cytometric analysis of apoptotic cell proportions in Colon Tumor 26 (CT26) and Lewis lung cancer cell line (3LL) cells after CEP55 knockdown.

We performed a comprehensive analysis of immune response data from seven clustered regularly interspaced short palindromic repeats (CRISPR) cohorts, encompassing 22,505 genes, which were stratified into 17 datasets based on cell types and treatment conditions. Genes were ranked by their mean *z* scores, identifying immune‐resistant genes at the top and immune‐sensitive genes at the bottom. Knockout of immune‐resistant genes may enhance antitumor immunity, while disruption of immune‐sensitive genes could diminish it. Figure 4A illustrates the gene ranking methodology. Next, we applied univariate Cox regression to the meta cohort, which includes the integrated training, validation, and test sets, to identify prognostic genes associated with a high iMLGAM score (*p* < 0.05). *Centrosomal Protein 55 (CEP55)* was ranked first among these genes based on the CRISPR data (Figure 4B). Analysis of TCGA pan‐cancer datasets showed that *CEP55* expression was upregulated in tumor samples compared to normal tissues (*p* < 0.05) (Figure S9A). To explore *CEP55*'s biological functions, we utilized TCGA pan‐cancer datasets and scRNA‐seq data, revealing its enrichment in cell cycle‐related pathways, particularly the G2M CHECKPOINT and E2F TARGETS (Figure S9B). In the pan‐cancer scRNA‐seq data set, we isolated tumor epithelial cells for dimensionality reduction and clustering, demonstrating *CEP55* expression in a subset of these cells (Figure S9C,D). GSEA further confirmed *CEP55*'s enrichment in cell cycle‐related pathways (Figure S9E). In conclusion, our findings highlight *CEP55* as a promising target for enhancing ICB therapy efficacy. By addressing tumor cell proliferation and immune evasion mechanisms, *CEP55*‐targeted strategies could potentially overcome existing limitations in immunotherapy and improve patient outcomes.

### *CEP55* knockdown attenuates cancer cell malignant phenotypes

To investigate the role of *CEP55* in cancer cell malignancy, tumor cells were transfected with control short hairpin RNA (shNC) or *CEP55*‐targeting shRNA (sh*CEP55*‐1, sh*CEP55*‐2) (Figure S10A). Transwell migration and invasion assays revealed that *CEP55* knockdown significantly inhibited the migration and invasion of Colon Tumor 26 (CT26) and Lewis lung cancer cell line (3LL) cells (*p* < 0.05) (Figure 4C,D, Figure S10B,C). Colony formation assays demonstrated a significant reduction in the ability of tumor cells to form colonies following *CEP55* knockdown (*p* < 0.05) (Figure 4E, Figure S10D). Flow cytometry analysis showed a marked increase in the proportion of apoptotic cells in CT26 and 3LL lines after *CEP55* knockdown (*p* < 0.05) (Figure 4F, Figure S10E). These results indicate that *CEP55* knockdown significantly inhibits tumor cell migration, invasion, and colony formation while promoting apoptosis. In summary, targeting *CEP55* markedly reduces the malignant behavior of cancer cells.

### *CEP55* downregulation enhances CD8 + T cell function and augments immunotherapy efficacy

**Figure 5:**
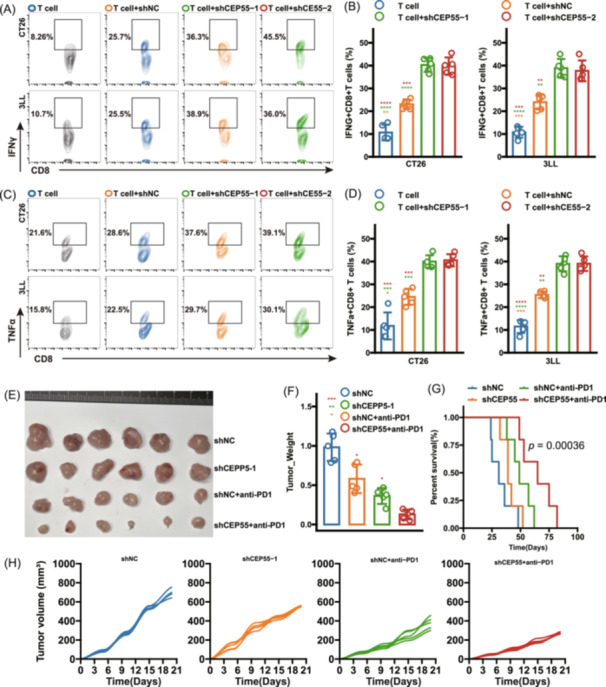
Impact of CEP55 knockdown on CD8^+^ T cell activity and tumor growth. (A) Flow cytometric analysis of IFN‐γ^+^ CD8^+^ T cells in CT26/3LL coculture systems with control short hairpin RNA (shNC) or *CEP55*‐targeting shRNA (sh*CEP55*) transfection. (B) Quantitative analysis of IFN‐γ^+^ CD8^+^ T cells in CT26 and 3LL coculture systems. (C) Flow cytometric analysis of TNF‐α^+^ CD8^+^ T cells in coculture systems. (D) Quantitative analysis of TNF‐α^+^ CD8^+^ T cells in CT26 and 3LL coculture systems. (E) Tumor images from 3LL subcutaneous mouse model under shNC, shCEP55, and shCEP55^+^anti‐PD1 treatments. (F) Tumor weight comparison across treatment groups in 3LL model. (G) Kaplan–Meier survival analysis of 3LL tumor‐bearing mice under shNC, shCEP55, and anti‐PD1 treatments. (H) Tumor growth curves (mm³) for different treatment groups in 3LL model.

This study investigated the impact of *CEP55* knockdown on CD8^+^ T cell immune function using coculture systems with CT26 and 3LL cancer cells. Tumor cells were transfected with either a nontargeting control shNC or *CEP55*‐targeting shRNAs and cocultured with CD8^+^ T cells. Results showed a significant increase in the percentage of IFN‐γ^+^ CD8^+^ T cells in the *CEP55* knockdown groups compared to controls (*p* < 0.05) (Figure 5A,B). Similarly, the percentage of TNF‐α^+^ CD8^+^ T cells was also significantly elevated in both CT26 and 3LL cells following *CEP55* knockdown (*p* < 0.05) (Figure 5C,D). Flow cytometry analysis revealed that *CEP55* knockdown in tumor cells decreased the expression of exhaustion markers PD‐1 and TIM‐3 on CD8^+^ T cells, indicating that reduced *CEP55* expression may reverse T cell exhaustion and restore functionality (*p* < 0.05) (Figure S10F,G). To assess the effects of *CEP55* knockdown on tumor growth and survival, we employed a mouse subcutaneous tumor model using 3LL cells (*n* = 5 per group). In this model, *CEP55* knockdown resulted in reduced tumor weight and volume compared to the control group (Figure 5E,F,H). Kaplan–Meier (K–M) survival analysis indicated that *CEP55* knockdown, especially in combination with anti‐programmed cell death protein 1 (anti‐PD1) therapy, significantly improved survival rates in tumor‐bearing mice (*p* < 0.05) (Figure 5G). These findings suggest that targeting *CEP55* may enhance the efficacy of immunotherapy.

## DISCUSSION

The identification of reliable predictive biomarkers remains essential for therapeutic decision‐making in immunotherapy, resource distribution optimization, and acceleration of both clinical investigations and regulatory endorsements. Although multiple biological indicators were previously investigated and implemented for immunotherapy outcome forecasting, their predictive capabilities were found to be inadequate [7, 21]. These limitations emphasized the critical requirement for alternative response signatures. In this investigation, which examined 1242 subjects with various malignancies, the iMLGAM score was developed and validated as a novel five gene‐pairs model for prognostic evaluation in patients receiving ICB therapy. This methodology represents the first pan‐cancer immunotherapeutic predictive system that utilizes ensemble learning through the integration of both gene‐pair analysis and GA approaches.

Our investigation presents multiple innovative elements and clinical applications. Initially, the research scope encompassed multiple malignancy categories, including NSCLC, melanoma, and RCC, which represent the predominant cancer types receiving ICB therapy in contemporary clinical practice. Subsequently, while numerous mRNA‐based indicators emerged as potential immunotherapeutic response predictors, the implementation of multi‐biomarker predictive systems required a thorough evaluation of factors affecting high‐throughput assay precision in clinical environments. Critical considerations included preanalytical (sample‐derived) and technical (platform‐derived) variation sources. Although tissue‐specific mRNA expression variations were traditionally normalized through reference gene incorporation and relative quantification protocols, the established risk‐score algorithms and threshold values for these mRNA signatures demonstrated limited compatibility across diverse analytical platforms, restricting their broad implementation [22]. To address these limitations, the IRGPs methodology was implemented, effectively eliminating batch variations. A previous study [12] demonstrated that a more precise prognostic assessment strategy through a gene pair analysis method, using PD‐L1 and CXCL10 as an example. Although these two genes were individually associated with favorable prognosis when analyzed separately, the PD‐L1 and CXCL10 gene pair is actually associated with significantly shortened survival. This finding stems from the complex interaction network of immune cells, highlighting the limitations of traditional single‐gene analysis methods. Thus, this approach enhanced ensemble learning model performance while offering multiple advantages: mitigation of immune cell co‐infiltration‐induced prognostic bias, capture of relative immune cell abundance patterns, and improved insights into cancer progression mechanisms. Gene pair‐based prediction systems exhibited enhanced stability across various sequencing platforms and specimen types, demonstrating superior translational potential. Additionally, these systems facilitated the detection of subtle immunological cellular variations while maintaining resistance to technical expression measurement fluctuations. This methodology provided enhanced robustness, biological interpretation capability, and clinical utility compared to individual gene expression analysis, particularly valuable for TIME investigation [12]. Furthermore, GA implementation optimized base learner selection within the ensemble framework, enhancing predictive accuracy. This integrated analytical approach demonstrated versatility beyond therapeutic response prediction, extending to various analytical applications incorporating gene expression and clinical data, including pan‐cancer prognostic evaluation. Finally, the iMLGAM scoring system presented significant clinical advantages. The methodology potentially protected nonresponsive individuals from immune‐related complications while facilitating the rapid transition to alternative therapeutic strategies. Considering the average treatment expenditure of $120,000 [23], the implementation of precise biomarker strategies could generate substantial cost reductions for interventions with predicted limited efficacy.

The comprehensive TCGA database was examined to characterize immunotherapeutic response patterns across malignancies. Analysis demonstrated that specimens with reduced iMLGAM scores exhibited an inflammatory immunological profile, marked by enhanced CD8^+^T lymphocyte infiltration and elevated immunogenicity. Through the application of immune infiltration parameters established by Danaher et al. and ssGSEA protocols, substantially increased immune cell infiltration was documented in the low iMLGAM cohort, validating robust antineoplastic immune activity. Previous investigations established positive associations between TIL density and immunological responses across diverse cancer types [24]. The population exhibiting reduced iMLGAM scores demonstrated not only enhanced cytotoxic T‐cell presence but also amplified expression of immune checkpoint molecules, encompassing PD‐L1, PD‐1, and CTLA‐4, compared to their high iMLGAM counterparts. This constellation of activated antitumor immunity, upregulated checkpoint molecule expression, and enhanced tumor immunogenicity was postulated to underlie the superior ICB therapeutic responses observed in subjects with low iMLGAM scores.

The robust predictive capabilities of the iMLGAM score necessitated the identification of associated molecular targets. CRISPR datasets were analyzed, with genes prioritized according to their sgRNA read logFCs between immune‐competent and immune‐deficient conditions. Subsequently, univariate Cox regression analysis was performed on the meta cohort to identify prognostic markers associated with elevated iMLGAM scores, leading to the identification of *CEP55* as a candidate for detailed examination. *CEP55*, a centrosome protein family constituent, was documented to regulate mitotic processes and cytokinesis. Its fundamental functions encompassed microtubule anchorage, protein polymerization facilitation, and spindle assembly regulation, collectively modulating cellular proliferation. Previous investigations demonstrated *CEP55* upregulation across multiple malignancies [25]. Enhanced *CEP55* expression was found to interact directly with PI3K p110 catalytic components, resulting in PI3K/AKT pathway activation. This activation promoted cellular transformation, proliferative activity, epithelial‐mesenchymal transition, and metastatic potential, thereby accelerating oncogenic progression [26]. Significantly, earlier functional genomic screening protocols identified *CEP55* among 182 genes whose modification influenced tumor susceptibility to T cell‐mediated cytotoxicity [27]. Within colorectal malignancies, elevated *CEP55* expression correlated with immunological exclusion and disease advancement, while its suppression enhanced antitumor immunity and ICB responsiveness [28]. Our study revealed that CEP55 downregulation substantially attenuated malignant cellular phenotypes while augmenting immunotherapeutic efficacy. These findings expanded previous observations by demonstrating that CEP55 suppression not only inhibited oncogenic potential but also enhanced immunotherapeutic responses, establishing CEP55 as a promising interventional target in cancer treatment.

Our study acknowledges several limitations. First, the 11 RNA‐seq cohorts analyzed represent a limited range of six cancer types, with the iMLGAM score's pan‐cancer predictive efficacy requiring further validation through prospective ICB trials. Second, some datasets lack critical clinical annotations, hindering comprehensive biomarker analyses. Third, our research primarily focused on validating CEP55 function in lung cancer, lacking extensive verification across other cancer types. Fourth, we currently lack large‐scale, multi‐center pan‐cancer cohort validation, limiting the model's generalizability. Fifth, this study failed to fully elucidate the precise molecular mechanism by which CEP55 regulates PD‐L1 expression. The specific signal transduction pathways and transcriptional regulatory mechanisms remain incompletely understood. Future research needs to conduct detailed protein interaction and signaling pathway analyses to reveal the molecular mechanisms of CEP55 regulation of PD‐L1 and its immune response modulation.

## CONCLUSION

The iMLGAM score represents a valuable predictive tool for clinical outcomes in cancer patients receiving ICB therapy. By integrating a gene‐pairing approach with advanced machine learning algorithms, this novel scoring system demonstrates improved predictive accuracy compared to existing biomarkers. A low iMLGAM score correlates with favorable immune activation patterns, while a high score indicates immune evasion mechanisms. Through comprehensive analysis, we identified CEP55 as a promising therapeutic target, with its knockdown significantly enhancing ICB therapy responses.

## METHODS

### Data collection and processing

We analyzed bulk transcriptomic profiles and clinical data from immunotherapy patients across 11 RNA‐seq datasets. This collection included four cohorts of melanoma (Łuksza [29], Gide [30], Riaz [31], and Hugo [32] cohorts), one data set for RCC (Bruan cohort [33]), one study on UC (Mariathasan cohort [34]), one cohort for GBM (Zhao cohort [33]), one data set for GC (Kim cohort [35]), and two cohorts of NSCLC (OAK and POPLAR [36] cohorts). The Hugo cohort comprised 27 pretreatment tumor specimens from 26 individuals, while the Zhao cohort [33] included 34 pretreatment samples from 17 patients; to maintain data independence, we randomly selected one tumor sample per patient from these datasets. We combined six ICB RNA‐seq cohorts to create a large data set (*n* = 1031), which included RCC (*n* = 172), UC (*n* = 298), melanoma (*n* = 244), and NSCLC (*n* = 317), merging Braun, Mariathasan, Łuksza, Gide, Riaz, and OAK cohorts. This data set was split into a training set (70%, *n* = 722) and a testing set (30%, *n* = 309). The remaining five ICB RNA‐seq cohorts were consolidated into an independent validation set (*n* = 211), as detailed in Table S3. To compare transcriptome signatures, we used the ComBat function from the sva R package [37] to adjust for batch effects.

Additionally, we obtained normalized transcriptomic and genomic data from 9,815 patients with complete survival information across 30 The Cancer Genome Atlas (TCGA) cohorts from the UCSC Xena database (https://xenabrowser.net) (Table S4). Three cancer types—diffuse large B cell lymphoma, acute myeloid leukemia, and thymoma—were excluded due to their high intrinsic immune cell content [38].

We also incorporated data from a pan‐cancer scRNA‐seq study [39], which included samples from 31 normal tissues, 54 adjacent tissues, and 148 tumors. We processed 829,521 cells using the Seurat R package [40], normalizing gene expression data with the LogNormalize method and identifying the 2000 most variably expressed genes for principal component analysis (PCA). Batch effects were managed using the Harmony R package [41]. The analysis pipeline included key steps such as NormalizeData, FindVariableFeatures, ScaleData, RunPCA, FindNeighbors, FindClusters, and RunUMAP. Individual cell annotations were obtained from the original study to ensure a thorough analysis of the cellular landscape.

Immune‐related genes were sourced from the ImmPort [42] database (https://immport.niaid.nih.gov) and the TimiGP R package [12] (Table S5).

### Clinical specimen collection and RNA sequencing

We established an in‐house ICB cohort comprising 14 patients diagnosed with metastatic or advanced NSCLC. In this study involving human subjects, informed consent has been obtained from the participants, and approval has been granted by the Ethics Committee of Changhai Hospital (No. CHEC2021091). These patients received treatment with anti‐PD‐1/PD‐L1 antibodies, either as monotherapy or in conjunction with anti‐CTLA‐4 antibodies, between January 2018 and October 2022. Ethical approval for the collection of NSCLC tissue samples before ICB therapy was obtained from the Medical Ethics Committee of Changhai Hospital. The samples were subsequently sent to Oncocare Inc. in Suzhou, China, for RNA sequencing.

### Clinical outcomes

The primary clinical evaluation endpoints were the objective response rate (ORR) and OS. The ORR was determined based on the response evaluation criteria in solid tumors (RECIST) v1.1 guidelines [43] for most cohorts, while the Hugo 2016 [32] was assessed using the immune‐related RECIST criteria. Patients were divided into two categories: responders, defined as those achieving complete response (CR) or partial response (PR), and non‐responders, which included individuals with stable disease (SD) or progressive disease (PD).

### Multicolor fluorescence IHC protocol

We utilized the Akoya OPAL Polaris 4‐Color Manual IHC Kit (NEL811001KT) for multicolor immunofluorescence staining of tissue specimens. The protocol began with baking the tissue slides to promote adhesion, followed by dewaxing to remove paraffin. Antigen retrieval was performed by heating the slides, and blocking of nonspecific antibody interactions was conducted. The slides were then incubated with primary antibodies: CD4 (1:500, Cat# Ab133616; Abcam), CD8A (1:2,000, Cat# ab217344; Abcam), and CD20 (1:100, Cat# ab64088; Abcam). Following this, incubation with HRP‐conjugated secondary antibodies was carried out. Signal amplification for each antigen was achieved through tyramide signal amplification, and nuclei were counterstained with DAPI. The staining process concluded with the mounting of coverslips for microscopy analysis, thereby enabling detailed visualization of cellular and molecular architectures within the tissue specimens.

### The framework of the iMLGAM score

The iMLGAM score was developed to predict clinical outcomes for cancer patients undergoing ICB therapy. This study employed feature selection and engineering techniques to identify crucial IRGPs using logistic regression and ROC analysis. The ABESS algorithm was utilized for optimized feature selection. Various machine learning methods, including Enet, RF, SVM, and KNN, were implemented to construct foundational learners, which were further refined through 10‐fold CV and grid search. A GA was employed to identify the optimal combination of learners for the iMLGAM score, with the AUC serving as the fitness function. The predictive capability of the iMLGAM score was evaluated using K–M analysis and ROC‐AUC. The source code and detailed parameters for the iMLGAM score can be accessed at the following GitHub repository: https://github.com/Yelab1994/iMLGAM.

### Evaluating iMLGAM score against other predictive gene signatures

To further evaluate the predictive capabilities of the iMLGAM score, we performed a comparative analysis with previously reported ICB signatures. This comparison encompassed 12 distinct signatures: CAF. Signature [43], INFγ. Sigature [44], chemokines. Sigature [45], Davoli_IS. Signature [46], CEP. Signature [44], CYT. Signature [47], TLS. Signature [48], TIDE. Signature [49], NLPR3. Signautre [50], Roh_IS. Signature [51], Ayers_expIS. Sigature [52], and IPS. Signautre [53]. The objective of this comprehensive comparison was to determine the relative effectiveness of the iMLGAM score among existing predictive models.

### Assessing tumor immune infiltration using *CIBERSORT*

*CIBERSORT* is a deconvolution algorithm that employs gene expression data alongside support vector regression to estimate the proportions of different cell types in mixed‐cell bulk cancer samples [54]. This method infers the relative abundances of 22 infiltrating immune cell types based on normalized gene expression profiles.

### Infiltration patterns of TILs and leukocytes

In the TCGA cohort, TIL levels were evaluated through two approaches: genomic assessment and analysis of H&E‐stained images. Data from Thorsson et al. and Saltz et al. [55, 56]. were used for these evaluations. Saltz et al. provided extensive TIL mappings for over 5000 H&E‐stained whole‐slide images from TCGA, utilizing deep learning techniques with convolutional neural networks to classify lymphocytes, thereby establishing a benchmark for TIL analysis. The genomic evaluation of TIL fraction combined two components: (1) the aggregated lymphocyte proportion in the immune compartment, determined by CIBERSORT, and (2) the leukocyte fraction derived from DNA methylation. Overall, this methodology enabled a comprehensive assessment of TILs across different cancer types in the TCGA data set.

### Assessing tumor immune infiltration scores

Immune infiltration scores were derived from a prior TCGA pan‐cancer study conducted by Danaher et al. [56]. These scores were calculated based on 60 specific marker genes and *single‐sample gene set enrichment analysis (ssGSEA)* method that classifies 14 distinct immune cell populations. The scores for each immune cell type were determined from the expression levels of these marker genes. The results exhibited high reproducibility and demonstrated strong concordance with findings obtained through IHC and flow cytometry techniques.

### Characterization of immune signatures

We acquired twenty‐nine classical immune signatures from the study by He et al. [57]. To quantify the enrichment levels of these signatures in each sample, we utilized the *GSVA* R package, employing the *ssGSEA* method [58].

### Assessing the abundance of fibroblasts using *MCPcounter*

The abundance of fibroblasts was estimated using the *MCPcounter* algorithm, implemented through the *MCPcounter* R package [59]. This algorithm offers a reliable method for quantifying immune and stromal cell populations across various tissues using transcriptomic data. Validated by both in vitro and ex vivo studies, *MCPcounter* outperforms earlier techniques by providing accurate estimates of cell abundances.

### Characterization of immunogenomic indicator

Immunogenomic indicators were derived from Thorsson et al.'s pan‐cancer immune landscape project [55]. The ITH score, defined as the subclonal genome fraction, was assessed using the *ABSOLUTE* algorithm, which models tumor copy number alterations and mutations. Copy number burden scores, specifically n_segs and frac_altered, represent the total number of segments in each sample's copy number profile and the proportion of bases deviating from baseline ploidy, respectively. Aneuploidy scores were calculated as the cumulative sum of amplified or deleted chromosomal arms. TCR and BCR diversity scores, including Shannon entropy and richness, were inferred from cancer RNA‐seq data. Together, these indicators offer valuable insights into the genomic characteristics of tumors and the diversity of immune receptors across various cancer types.

### Unraveling genomic mutational patterns

The *maftools* R package was utilized to perform nonnegative matrix factorization analysis of mutations categorized by 96 trinucleotide contexts in pan‐cancer specimens from TCGA. The resulting mutational profile was subsequently compared to the Catalogue of Somatic Mutations in Cancer using cosine similarity.

### Oncogenic pathway enrichment analysis

Ten canonical oncogenic pathways, comprising 187 oncogenes, were obtained from the study by Sanchez‐Vega et al. [60]. To assess the activity of these pathways in each sample, we utilized the ssGSEA method within the *GSVA* R package [58].

### Deciphering the impact of copy number variations

*GISTIC 2.0*, an advanced computational tool [61], was employed to investigate significant deletion or amplification events in genomic regions. This program identifies somatic copy number alterations by analyzing both the amplitude and frequency of observed alterations. Using *GISTIC 2.0*, we were able to identify recurrent genomic changes that may be critical in the development and progression of cancer across various tumor types.

### Functional enrichment analysis

Functional enrichment analysis and clustering of identified biological processes were carried out using the R package *clusterProfiler* [62].

### CRISPR screening data

To investigate potential therapeutic targets of iMLGAM score, we aggregated data from seven published CRISPR/Cas9 screening studies that evaluated the individual effects of each gene knockout on tumor immunity, including Freeman [63], Kearney [64], Manguso [65], Pan [66], Patel [67], Vredevoogd [68], and Lawson [27] (Table S6). The first six CRISPR studies were previously curated by Fu et al. [49]. In addition to Fu et al., we further compiled another CRISPR cohort from Lawson et al. [27]. To explore potential therapeutic targets related to the iMLGAM score, we aggregated data from seven CRISPR/Cas9 screening studies assessing gene knockout effects on tumor immunity (Table S6). The first six studies were curated by Fu et al. [49], and we added data from Lawson et al. [27]. These studies were organized into 17 datasets based on cell lines and treatment conditions, encompassing melanoma, breast, colon, and renal cancer. We aimed to identify genes that could enhance lymphocyte‐mediated cancer killing and influence immunotherapy responses. The CRISPR screening involved genome‐wide knockout in cancer cell lines, followed by RNA‐Seq to measure sgRNA abundance. Log‐fold changes of sgRNA reads assessed knockout effects on cancer fitness under immune pressure. Normalized z‐scores from these changes facilitated cross‐study comparisons, with lower *z*‐scores indicating better immune responses. Genes were ranked by average *z*‐scores across the datasets, with top‐ranked genes classified as immune resistant.

### Cell culture

The 3LL and CT26 cells were sourced from the China Center for Type Culture Collection. LLC cells were cultured in DMEM, whereas CT26 cells were maintained in RPMI 1640 medium. Both media were supplemented with 10% fetal bovine serum (FBS), 2 mM l‐glutamine, 1 mM pyruvate, 100 U/mL penicillin, and 100 µg/mL streptomycin.

### Western blot

Cells were lysed on ice for 20 min in a buffer containing 1× RIPA, 1× phosphatase inhibitor, and 1× protease inhibitor (Thermo Fisher Scientific). The lysate was centrifuged at 1200×*g* for 5 min at 4°C, and the supernatant was collected. Total protein concentration was assessed using the BCA Protein Assay Kit (Thermo Fisher Scientific), and 40 µg of protein was loaded per well for SDS gel electrophoresis. Proteins were separated at 120 V for 1 h and transferred to a PVDF membrane (Thermo Fisher Scientific) at 15 V for 30 min. The membrane was stained with Licor Revert Total Protein Stain for visualization. After washing with TBST and blocking with 5% bovine serum albumin, primary antibodies were incubated overnight at 4°C. The following day, the membrane was washed again and incubated with a secondary antibody at room temperature for 1 h. Membrane imaging was conducted using Amersham™ ECL Select (Cytiva). Primary antibodies were diluted at 1:125 for CEP55 (Santa Cruz) and 1:1500 for Beta‐Actin (Cell Signaling Technology).

### Lentiviral stable transfection cell line construction

Stable CEP55 knockdown cell lines were generated in 3LL and CT26 cells using lentiviral transduction. HEK293T cells were first transfected with lentiviral packaging plasmids (psPAX2 and pMD2.G) along with either CEP55‐targeting shRNA or shNC. After 48 h, the lentiviral supernatants were collected and used to infect 3LL and CT26 cells with polybrene (8 μg/mL) to enhance infection efficiency. The target sequences were: sh#1: CAGCATCAACTCTATGTGATT and sh#2: CGTTTAGAACTCGATGAATTT. Following 24 h of incubation, the medium was replaced with fresh DMEM, and the cells were allowed to recover for 48 h. Puromycin (2–10 μg/mL, optimized for each cell line) was then added for selection, maintained for 5–7 days to establish stable transfected cells. CEP55 knockdown efficiency was validated by qPCR and Western blot analysis. These stable cell lines were subsequently used in functional experiments to evaluate the role of CEP55 in tumor immunity.

### Clone formation

Dilute the single‐cell suspension and seed 500 cells per well in a six‐well plate. Incubate for 10 days at 37°C with 5% CO_2_. Afterward, wash with PBS, fix with 4% paraformaldehyde for 30 min, and stain with crystal violet for 20 min. Rinse with running water, then photograph and count the colonies formed.

### Transwell

To assess cell migration, dilute the tumor cells to a single‐cell suspension and add 2 × 10^5^ cells to the upper chamber of the Transwell, with 600 μL of serum‐free medium in the lower chamber. Incubate for 36 h at 37°C with 5% CO_2_. After incubation, remove non‐migrated cells from the upper chamber with a cotton swab, fix the remaining cells with 4% paraformaldehyde for 30 min, and stain with 0.1% crystal violet for 20 min. Wash three times with PBS and count the cells that migrated to the lower chamber under a microscope. For the invasion assay, dilute Matrigel per the manufacturer's instructions and coat the upper chamber, incubating at 37°C for 1 h to solidify. Repeat the same procedure as above: add 2 × 10^5^ tumor cells, incubate for 36 h, remove non‐invaded cells, fix, stain, wash, and count the cells that invaded the lower chamber.

### Flow cytometry apoptosis assay

After collecting and washing the cells with pre‐cooled PBS, resuspend them in Annexin V binding buffer (approximately 1 × 10^6^ cells per experiment). Add 5 µL of Annexin V‐FITC and 5 µL of PI to the suspension, and incubate in the dark at room temperature for 15 min. Analyze the stained cells for apoptosis using a flow cytometer and *FlowJo* software.

### Coculture of CD8^+^ T cells and tumor cells

Cells were isolated from the spleens of wild‐type C57BL/6 mice and filtered through a 70 µm filter to create a single‐cell suspension. After lysing red blood cells, CD8^+^ T lymphocytes were isolated using an immune magnetic bead sorting kit (Cat # 480008, BioLegend). T cells were counted and seeded into 12‐well plates coated with 2.0 mg/mL anti‐CD3 (clone 145–2C11; BioLegend) and 3 mg/mL anti‐CD28 (clone 37N; BioLegend) antibodies in complete RPMI 1640 medium supplemented with 50 mM β‐mercaptoethanol and 10 mM HEPES. Following 48 h of activation, T cells were cocultured with tumor cells from either knockdown shNC or shCEP55. After an additional 48 h, T cells were collected for functional assays, which were analyzed using *FlowJo* software.

### Establishment of mouse subcutaneous tumor model

The procedure related to animal subjects was approved by the Ethics Committee of the Tianjin Medical University Cancer Institute and Hospital (No. 2023078). All mice were housed in the animal facility of Tianjin Medical University Cancer Institute and Hospital under specific‐pathogen‐free conditions. Male wild‐type C57BL/6 mice (6–8 weeks old) were placed in a sterile environment. Tumor cells were resuspended in PBS at a concentration of 5 × 10^6^ cells/mL, and each mouse was subcutaneously inoculated with 100 μL of the suspension (5 × 10^5^ cells) on the right dorsal flank. When tumor volumes reached approximately 100 mm³, mice were randomly grouped and treated by intraperitoneal injection with mouse PD‐1 monoclonal antibody (BioXcell; BE0146) at a dose of 100 mg per mouse dissolved in 100 μL d‐PBS buffer. Tumor size was measured with calipers every 2–3 days, and volume was calculated using the formula (volume = length × width²/2). Mice had free access to food and water and were kept in a suitable temperature environment. Upon reaching the predetermined tumor size or at the experiment's conclusion, mice were euthanized by cervical dislocation, and tumor tissues were collected for further analysis.

### Flow cytometry for tumor‐bearing mice

After tumor inoculation, tumor‐bearing mice were killed, and 3LL tumor samples were collected and chopped. The samples were incubated with a digestive solution at 37°C for 1 h and filtered through a 70 µm filter to obtain a single‐cell suspension. Red blood cells were lysed on ice with lysis buffer for 5 min, then washed and resuspended in pre‐cooled phosphate‐buffered saline (PBS). T cells were stimulated for 4 h at 37°C in a CO_2_ incubator with complete RPMI 1640 medium containing 10% FBS, 1.5 μg/mL monensin, 100 ng/mL PMA, and 1 μg/mL ionomycin. The cell suspension was washed, blocked with CD16/32, and stained for viability on ice in the dark for 20 min. After washing, cells were stained for 25 min on ice with primary antibodies against CD45 (APC‐CY7), CD8 (FITC), CD3 (PE‐CY5.5), PD‐1 (PB450), and TIM‐3 (BV605). Following staining, cells were fixed with 1% paraformaldehyde for 20 min, permeabilized, and stained intracellularly with granzyme B (GZMB, PerCP‐CY5.5), interferon (IFN)‐γ (APC), and tumor necrosis factor (TNF)‐α (PE/Cyanine7). Finally, cells were analyzed using a Beckman DxFLEX flow cytometer, with data processed using *FlowJo* software.

### Statistical analysis

Pearson's or Spearman's coefficients assessed correlations between variables. A *t*‐test analyzed group differences for normally distributed variables; otherwise, the Mann–Whitney *U* test was used. Categorical variable differences were evaluated with Pearson's chi‐squared test or Fisher's exact test. K–M analysis generated survival curves, and the log‐rank test assessed significance. ROC analysis determined optimal threshold values, while multivariate logistic regression evaluated independent risk factors for clinical response. A *p*‐value of less than 0.05 was considered statistically significant. All analyses were two‐sided and performed using *R* software (version 4.3.1).

## AUTHOR CONTRIBUTIONS

**Bicheng Ye**: Writing—original draft; writing—review and editing; methodology; software. **Jun Fan**: Writing—original draft; writing—review and editing; methodology. **Lei Xue**: Writing—original draft; writing—review and editing. **Yu Zhuang**: Writing—original draft; writing—review and editing. **Peng Luo**: Software. **Aimin Jiang**: Software. **Jiaheng Xie**: Software. **Qifan Li**: Methodology. **Xiaoqing Liang**: Methodology. **Jiaxiong Tan**: Methodology. **Songyun Zhao**: Methodology. **Wenhang Zhou**: Writing—review and editing; conceptualization. **Chuanli Ren**: Writing—review and editing; conceptualization. **Haoran Lin**: Conceptualization; writing—review and editing; writing—original draft. **Pengpeng Zhang**: Conceptualization; writing—review and editing.

## CONFLICT OF INTEREST STATEMENT

The authors declare no conflicts of interest.

## ETHICS STATEMENT

The procedure related to human subjects was approved by the Ethics Committee of the Changhai Hospital (No. CHEC2021091). The procedure related to animal subjects was approved by the Ethics Committee of the Tianjin Medical University Cancer Institute and Hospital (No. 2023078).

## Supporting information


**Figure S1:** Survival Analysis and Predictive Performance.
**Figure S2:** Distribution and Predictive Performance of integrated Machine Learning and Genetic Algorithm‐driven Multiomics analysis (iMLGAM) Score for immune checkpoint blockade (ICB) Therapy Response Across Multiple Cancer Types.
**Figure S3:** Assessment of iMLGAM score in Multiple Independent Immunotherapy Cohorts.
**Figure S4:** Pan‐Cancer Analysis of iMLGAM Score.
**Figure S5:** Comparative Analysis of Immune Signatures, Cytolytic Activity, and Tumor Microenvironment Features Between iMLGAM Score Groups.
**Figure S6:** Molecular and Immunological Landscape Analysis.
**Figure S7:** Copy Number Alterations in High and Low iMLGAM Score Groups.
**Figure S8:** Quantitative analysis of immune cell populations.
**Figure S9:** Pan‐Cancer Analysis of Centrosomal Protein 55 (CEP55) Expression and Its Associated Molecular Signatures.
**Figure S10:** Functional Characterization of CEP55 Knockdown Effects.

**Table S1:** List of bulk RNA sequencing (bulk RNA‐seq) immunotherapy datasets.
**Table S2:** List of pan‐cancer transcriptomic datasets in TCGA.
**Table S3:** List of Immune‐related genes.
**Table S4:** List of Clustered Regularly Interspaced Short Palindromic Repeats (CRISPR) datasets.
**Table S5:** List of key immune‐related gene pairs (IRGPs).
**Table S6:** List of basic learners.

## Data Availability

Public data used in this study are available in TCGA and GEO. Other datasets were obtained from published literature, as described in the Methods section. The analytic processes, code and data of this study are embedded in the R package 'iMLGAM' and are available at https://github.com/Yelab1994/iMLGAM. Public data used in this study are available in TCGA and GEO. Other datasets were obtained from published literature, as described in the Methods section. The analytic processes, code and data of this study are embedded in the R package “iMLGAM” and are available at https://github.com/Yelab1994/iMLGAM. Supplementary materials (figures, tables, graphical abstract, slides, videos, Chinese translated version, and update materials) may be found in the online DOI or iMeta Science http://www.imeta.science/.
