# Supplementary material for: iMLGAM: Integrated Machine Learning and Genetic Algorithm‐driven Multiomics analysis for pan‐cancer immunotherapy response prediction
Source: Imeta. 2025 Mar 8;4(2):e70011. doi: 10.1002/imt2.70011 (PMC11995183; doi:10.1002/imt2.70011)

**Supporting information to ：**

**iMLGAM: integrated Machine Learning and Genetic Algorithm-driven Multiomics analysis for pan-cancer immunotherapy response prediction**

**Running title**:Pan-cancer immunotherapy prediction by iMLGAM

Bicheng Ye^1#^, Jun Fan^2#^, Lei Xue^2#^, Yu Zhuang^3, 4#^, Peng Luo^5^, Aimin Jiang^6^, Jiaheng Xie^7^, Qifan Li^8^, Xiaoqing Liang^9^, Jiaxiong Tan^10^, Songyun Zhao^11^, Wenhang Zhou^12*^, Chuanli Ren^13*^, Haoran Lin^2*^, Pengpeng Zhang^2, 10*^

^1^ Liver Disease Center of Integrated Traditional Chinese and Western Medicine, Department of Radiology, Zhongda Hospital, Medical School, Southeast University, Nurturing Center of Jiangsu Province for State Laboratory of AI Imaging & Interventional Radiology (Southeast University), Nanjing 210009 , China

^2^ Department of Thoracic Surgery, The First Affiliated Hospital of Nanjing Medical University, Nanjing 210029, China

^3^ Department of Thoracic surgery, Nanjing Chest Hospital, Nanjing 210029, China

^4^ Afliated Nanjing Brain Hospital, Nanjing Medical University, Nanjing 210024, China

^5^ Department of Oncology, Zhujiang Hospital, Southern Medical University, Guangzhou 510282, China

^6^ Department of Urology, Changhai Hospital, Naval Medical University (Second Military Medical University), Shanghai 200433, China

^7^ Department of Plastic Surgery, Xiangya Hospital, Central South University, Changsha 410028, Chinaa

^8^ Department of Thoracic Surgery, The First Affiliated Hospital of Soochow University, Suzhou 215006, China

^9^ Chongqing Key Laboratory of Molecular Oncology and Epigenetics, The First Affiliated Hospital of Chongqing Medical University, Chongqing 400016, China

^10^ Department of Lung Cancer, Tianjin Lung Cancer Center, National Clinical Research Center for Cancer, Key Laboratory of Cancer Prevention and Therapy, Tianjin's Clinical Research Center for Cancer, Tianjin Medical University Cancer Institute and Hospital, Tianjin 300060, China

^11^ Department of Plastic Surgery, The First Affiliated Hospital of Wenzhou Medical University, Wenzhou 325000, China

^12^ Department of Oncology, the Affiliated Huai'an Hospital of Xuzhou Medical University, the Second People's Hospital of Huai'an, Huai'an 223002, China

^13^ Department of Laboratory Medicine, Northern Jiangsu People's Hospital Affiliated to Yangzhou University, Yangzhou 225009, China

^#^These authors contributed equally: Bicheng Ye, Jun Fan, Lei Xue, Yu Zhuang

*Correspondence:huaianzhouwenhang@163.com (Wenhang Zhou); 18051061089@yzu.edu.cn (Chuanli Ren); linhaoran@jsph.org.cn (Haoran Lin); zpp19940120@tmu.edu.cn (Pengpeng Zhang);

**Supplementary figures:**

**Figure S1.** Survival Analysis and Predictive Performance. **A-C.** K-M survival analysis for iMLGAM score in the training (A), validating (B), and testing (C) cohorts. **D-F.** AUC values of the iMLGAM score in predicting 2 and 3-year survival rates in the training (D), validating (E), and testing (F) cohorts.

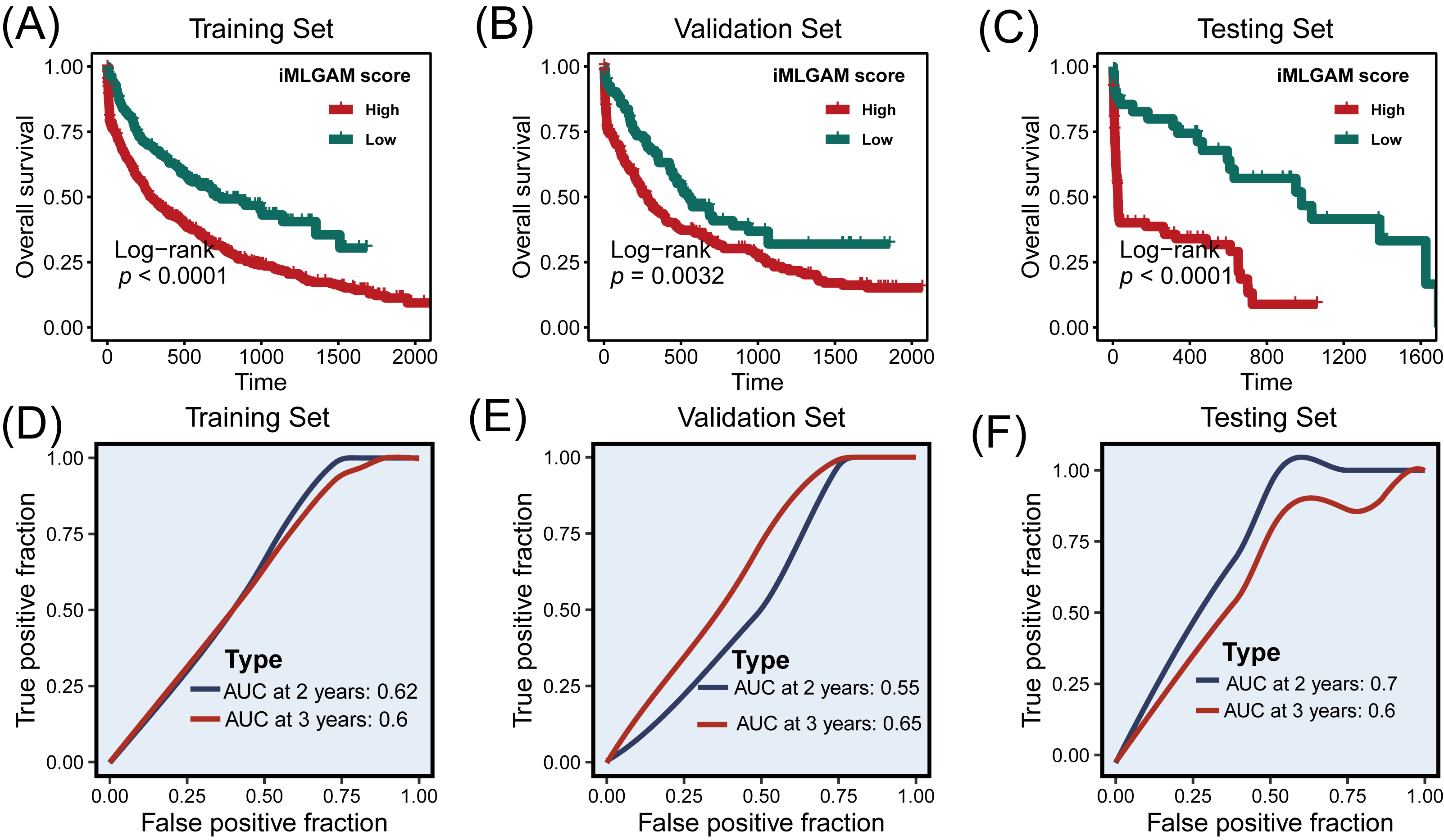


**Figure S2.** Distribution and Predictive Performance of iMLGAM Score for ICB Therapy Response Across Multiple Cancer Types. The distribution of iMLGAM score between responders and nonresponders of ICB therapy in RCC (A), NSCLC (C), melanoma (E), STAD (G), UC (I), and GBM patients (K). ROC curves of iMLGAM score to predict the benefits of ICB therapy in RCC (B), NSCLC (D), melanoma (F), STAD (H), UC (L), and GBM patients (L) . NS, no significant; **p* < 0.05; ***p* < 0.01; ****p* < 0.001.


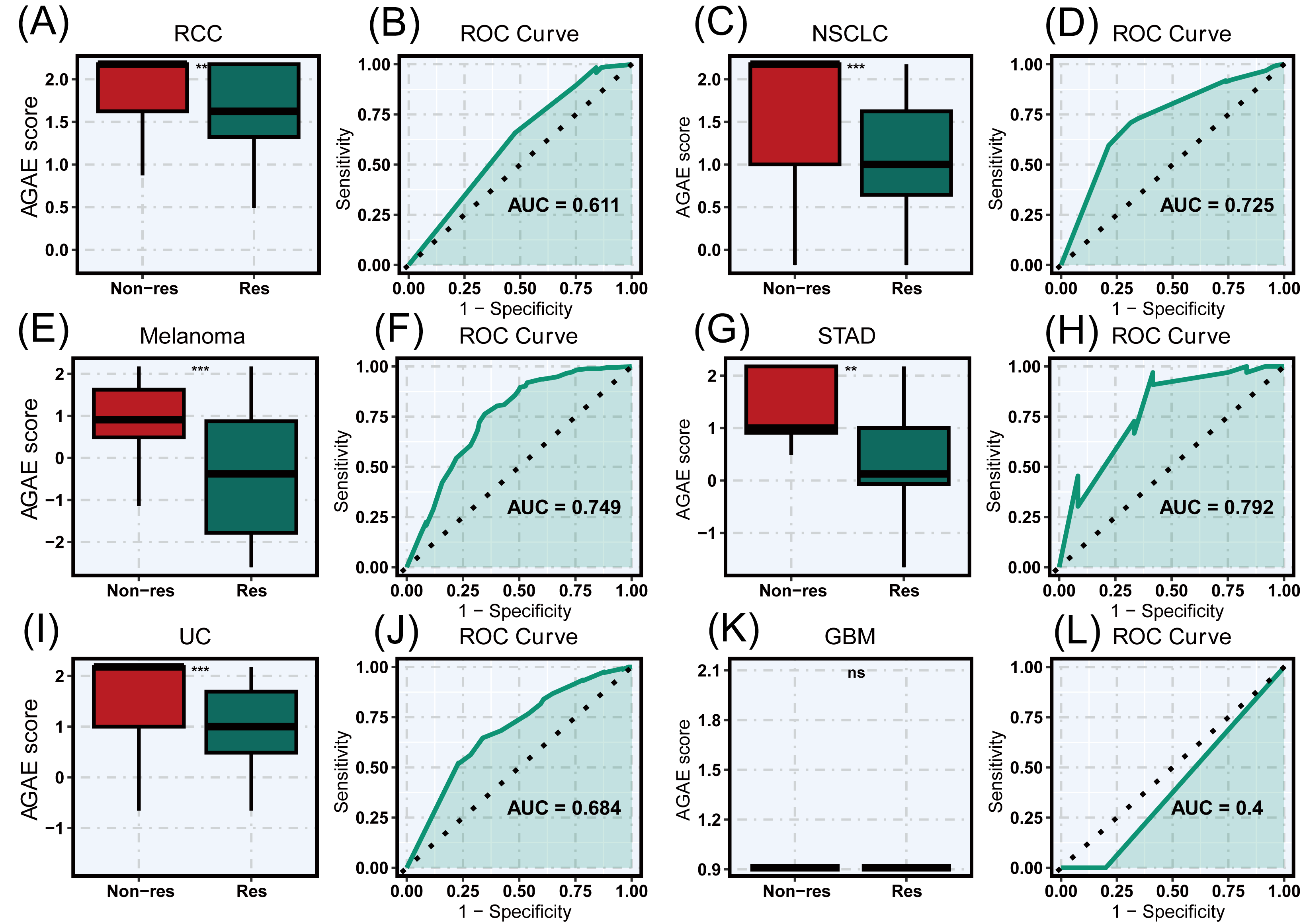


**Figure S3.** Assessment of iMLGAM score in Multiple Independent Immunotherapy Cohorts. **A-D.** Comparative analysis of iMLGAM score versus TMB and PD-L1 expression using receiver operating characteristic ROC curves across multiple independent cohorts: Mariathasan cohort (A), Braun cohort (B), OAK cohort (C), and Riaz cohort (D). **E-H.** Multivariate logistic regression models evaluating the independent predictive value of iMLGAM score after adjusting for clinicopathological variables in the Mariathasan (E), Braun (F), OAK trial (G), and Riaz (H) cohorts.


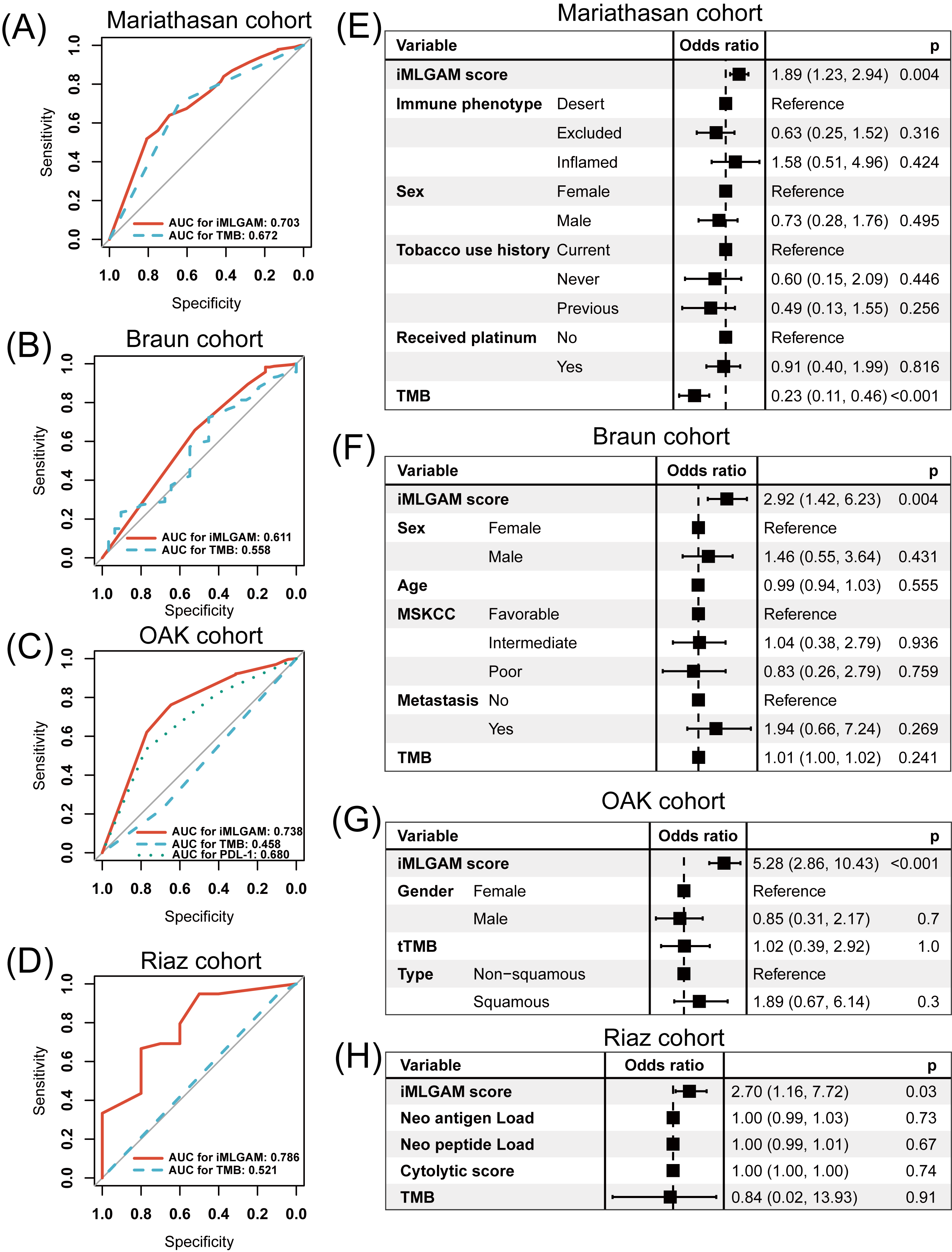


**Figure S4.** Pan-Cancer Analysis of iMLGAM Score. **A.** Distribution of iMLGAM scores across 30 cancer types in TCGA database. **B.** Differential analysis of leukocyte fractions between high and low iMLGAM score groups based on DNA methylation profiles. **C.** Comparative analysis of lymphocyte populations between high and low iMLGAM score groups using CIBERSORT algorithm on RNA-sequencing data. **D.** Quantitative comparison of tumor-infiltrating lymphocyte (TIL) fractions between high and low iMLGAM score groups based on molecular estimates from cancer genomics data. **E.** Analysis of spatial TIL distribution between high and low iMLGAM score groups derived from diagnostic H&E image processing. **F.** Differential analysis of CD8+ T cell abundance between high and low iMLGAM score groups quantified by CIBERSORT algorithm using RNA-sequencing data. **G.** Comprehensive comparison of 14 immune cell populations between high and low iMLGAM score groups using Danaher method on RNA-sequencing data. **H.** Comparative analysis of 29 immune signatures between high and low iMLGAM score groups using single-sample Gene Set Enrichment Analysis (ssGSEA) on RNA-sequencing data. **I.** Unsupervised clustering analysis of TCGA cohort based on 29 immune signatures revealing two distinct immune subtypes. **J.** Distribution analysis of high versus low immune infiltration patterns between high and low iMLGAM score groups based on 29 immune signatures.note: **p* < 0.05; ****p* < 0.001.


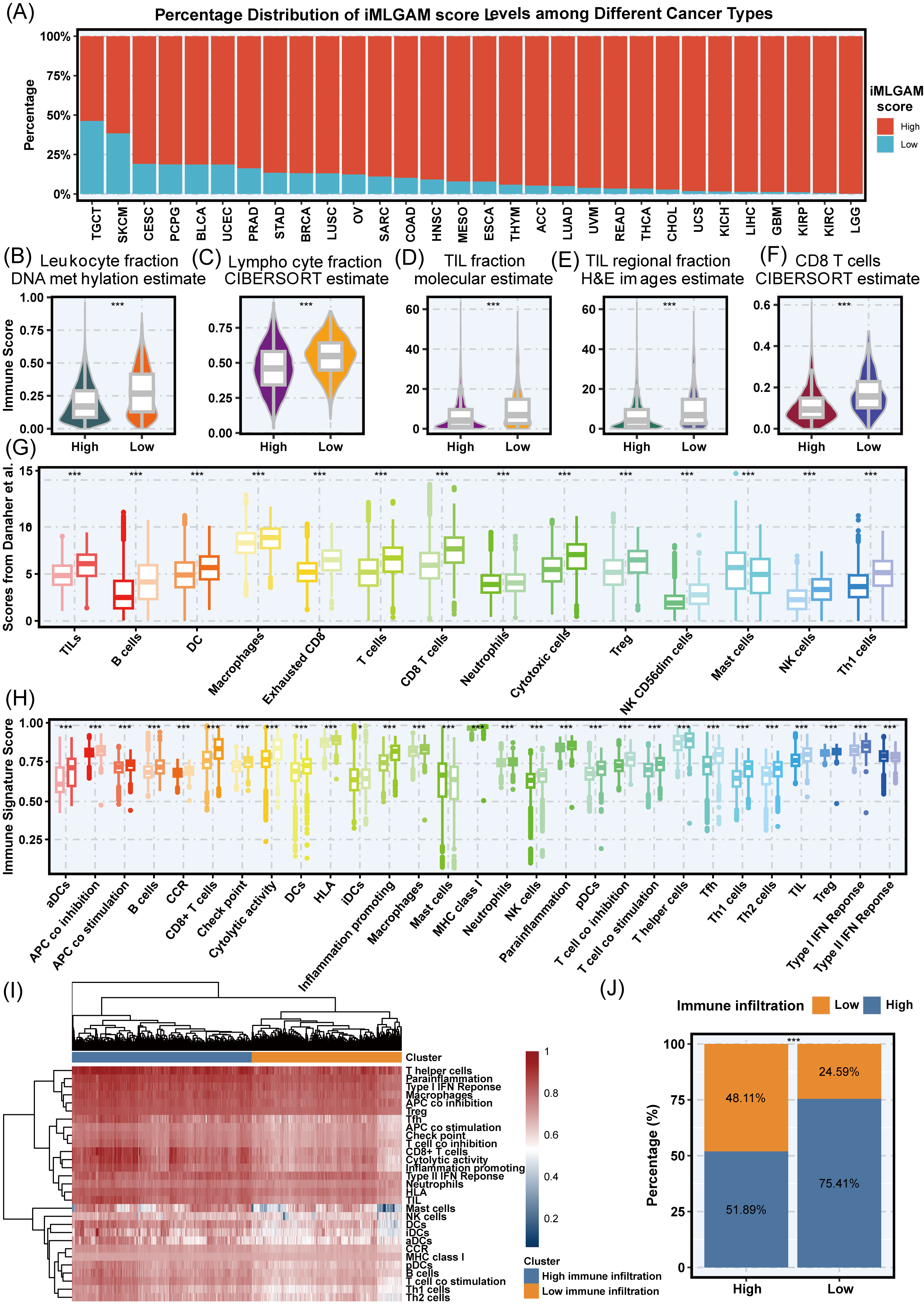


**Figure S5.** Comparative Analysis of Immune Signatures, Cytolytic Activity, and Tumor Microenvironment Features Between iMLGAM Score Groups. **A.** Comparative analysis of correlation coefficients among 29 immune signatures between high and low iMLGAM score groups. **B.** Quantitative comparison of cytolytic activity scores between high and low iMLGAM score groups. **C.** Differential analysis of stromal fibroblast abundance between high and low iMLGAM score groups. **D.** Comparative analysis of chemokine expression profiles between high and low iMLGAM score groups. NS, no significant; **p* < 0.05; ***p* < 0.01; ****p* < 0.001.


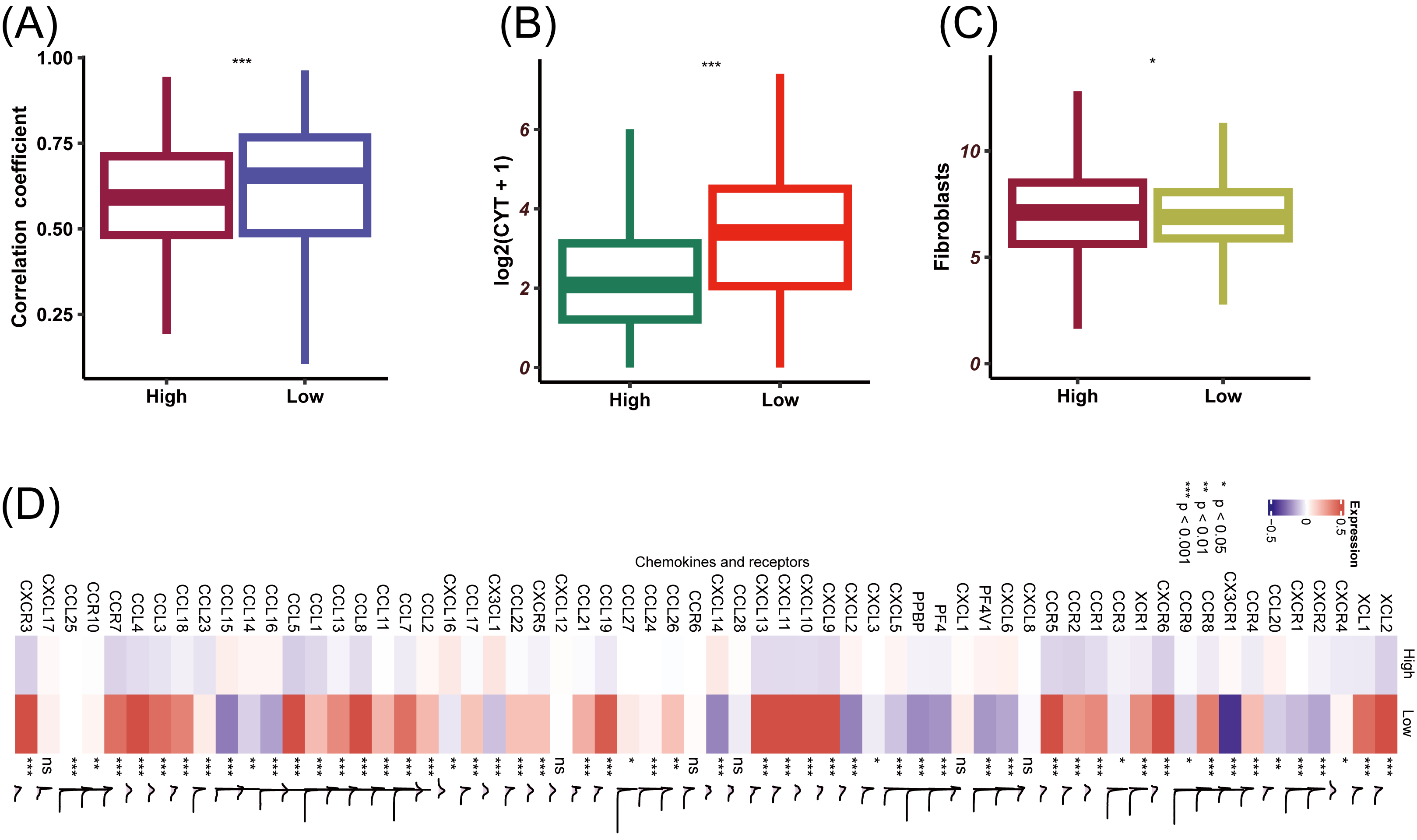


**Figure S6.** Molecular and Immunological Landscape Analysis. **A.** Differential analysis of immunogenomic parameters between high and low iMLGAM score groups. **B.** Characterization of mutational activities across four distinct mutational signatures. **C.** Comparative assessment of four mutational signature profiles between high and low iMLGAM score groups. **D.** Differential enrichment analysis of 10 oncogenic signaling pathways between high and low iMLGAM score groups. **E.** Expression profile analysis of immune regulatory molecules between high and low iMLGAM score groups, including: MHC molecules, Costimulatory molecules and Coinhibitory molecules. NS, no significant; **p* < 0.05; ***p* < 0.01; ****p* < 0.001.


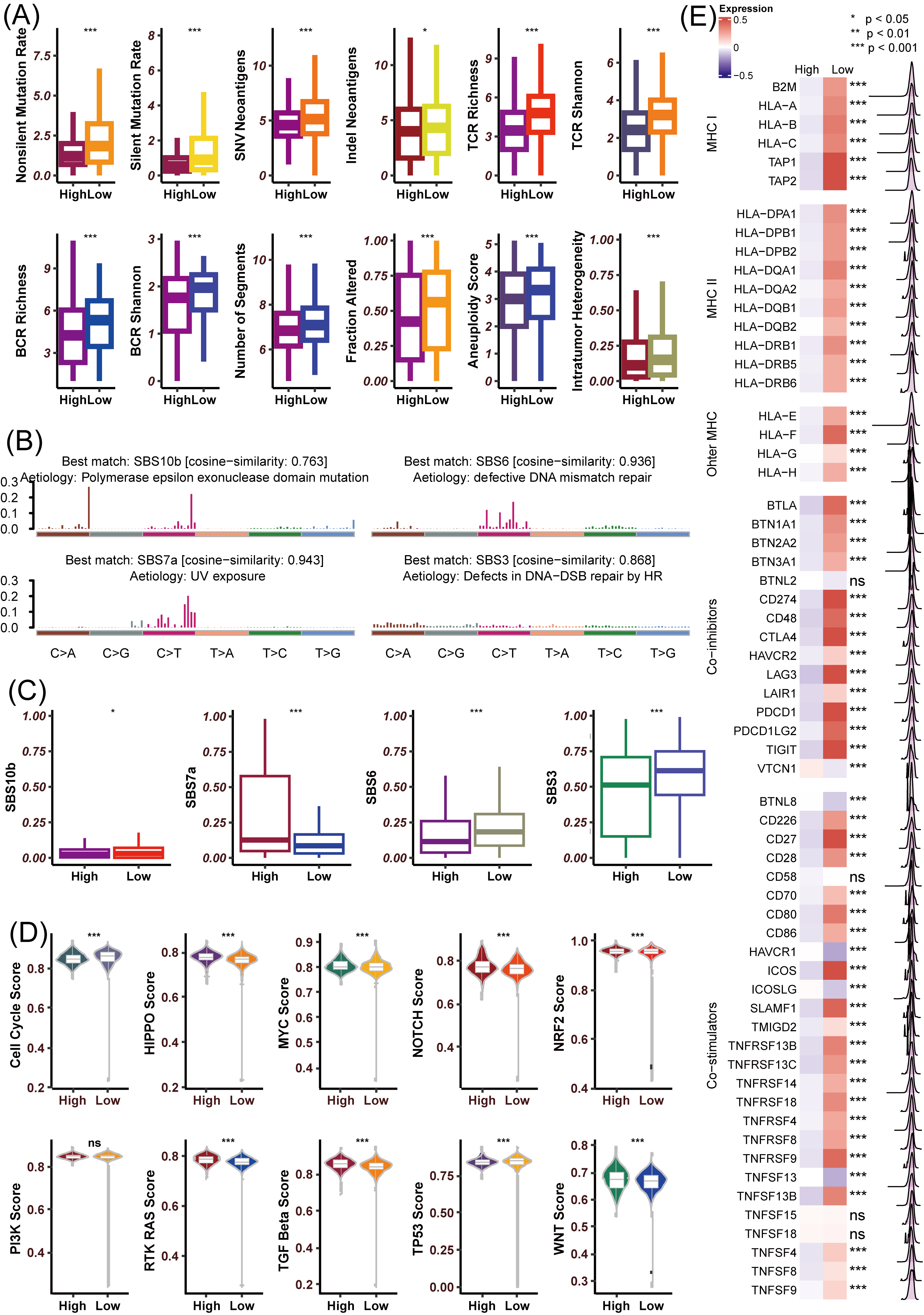


**Figure S7.** Copy Number Alterations in High and Low iMLGAM Score Groups. **A.** Copy number profiles comparing high iMLGAM score (above) and low iMLGAM score (below) groups. Red indicates gains; green indicates losses. **B.** Focal amplification (left) and deletion (right) peaks by cytobands identified in high iMLGAM score group. **C.** Focal amplification (left) and deletion (right) peaks by cytobands identified in low iMLGAM score group. **D.** Venn diagrams showing significantly amplified genes in high and low iMLGAM score groups. Numbers in overlapping regions represent shared genes between groups. **E.** Cluster analysis of top 10 biological processes in high iMLGAM score (left) and low iMLGAM score (right) groups. **F.** Circular plot depicting 10 biological processes and corresponding enriched genes in high iMLGAM score (left) and low iMLGAM score (right) groups. **G.** Comparison of PD-L1 and PD-1 mRNA expression between high and low iMLGAM score groups in TCGA cohort. NS, no significant; **p* < 0.05; ***p* < 0.01; ****p* < 0.001.


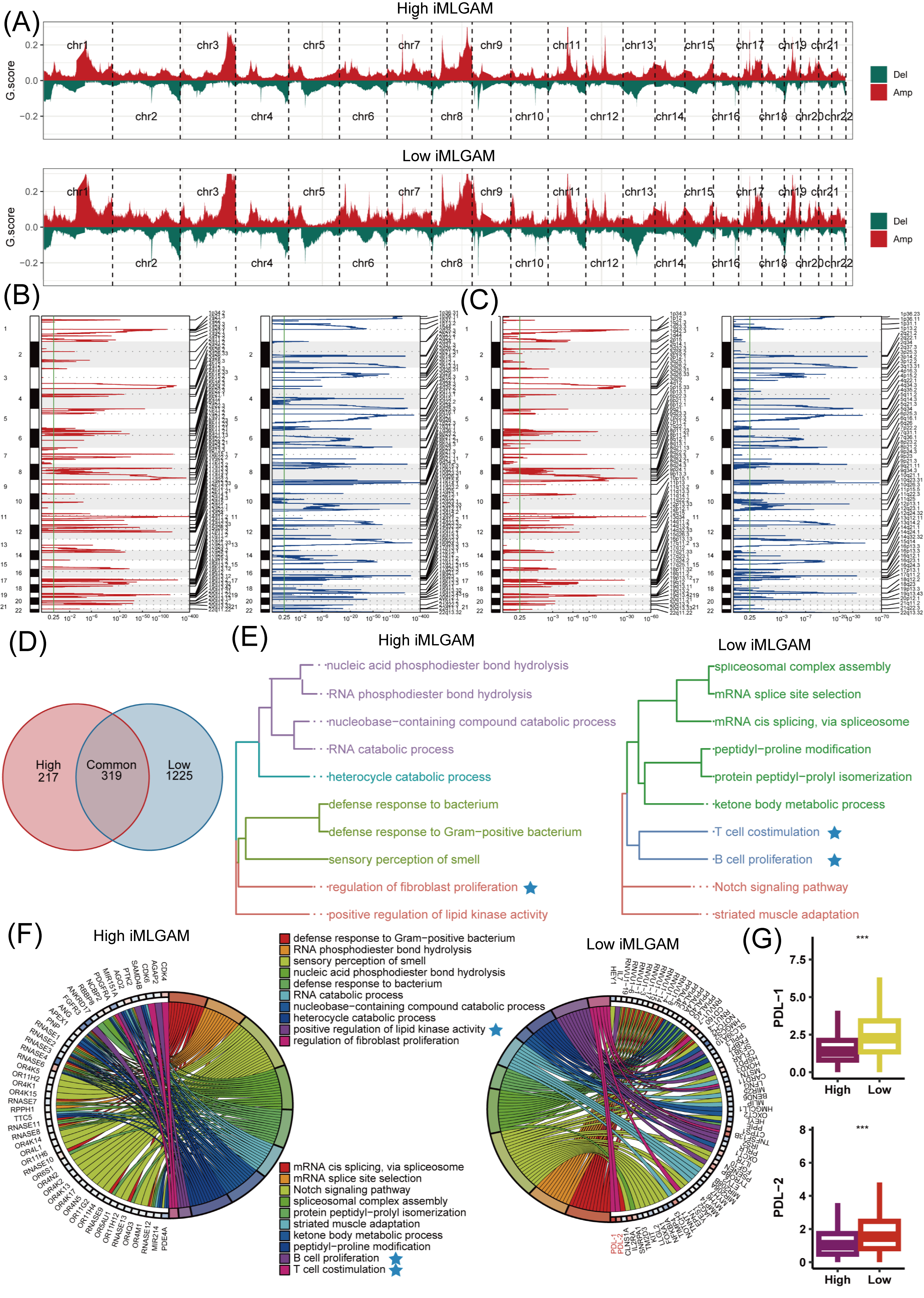


**Figure S8.** Quantitative analysis of immune cell populations. **A.** The ratio of CD20+ B cells is significantly higher in the iMLGAM-Low group compared to the iMLGAM-High group (****p < 0.0001). **B.** The ratio of CD8+ T cells also shows a significant increase in the iMLGAM-Low group compared to the iMLGAM-High group (****p < 0.0001). Data are presented as mean ± SEM, with individual data points indicated. Statistical significance was determined using an appropriate statistical test.


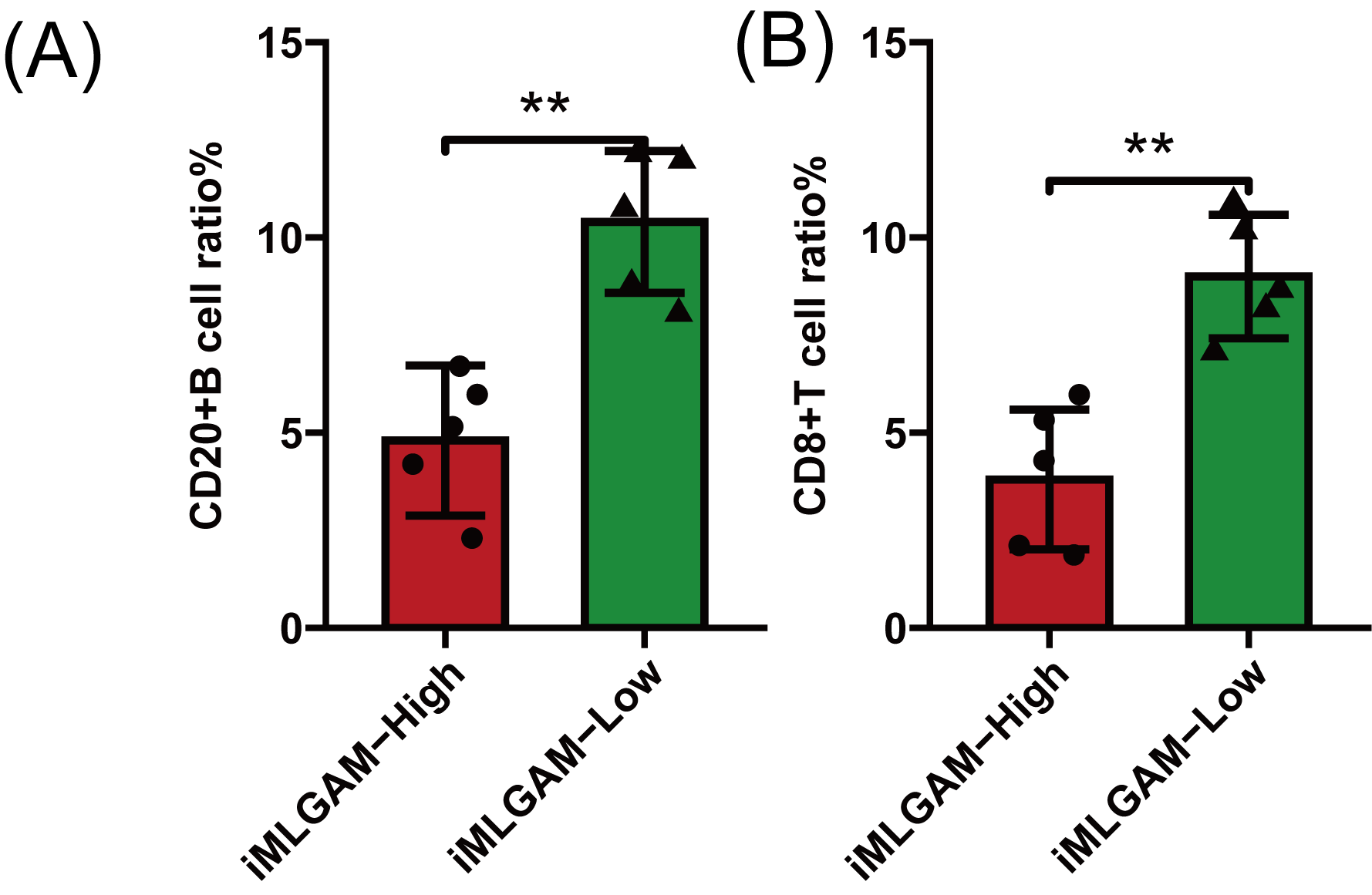


**Figure S9.** Pan-Cancer Analysis of CEP55 Expression and Its Associated Molecular Signatures. **A.** CEP55 expression profile across TCGA cancer types. **B.** GSEA of CEP55 using bulk RNA-seq data. **C.** UMAP visualization of epithelial cell populations. **D.** UMAP visualization of CEP55 mRNA expression distribution. **E.** GSEA of CEP55 using single-cell RNA-seq data. NS, no significant; **p* < 0.05; ***p* < 0.01; ****p* < 0.001.


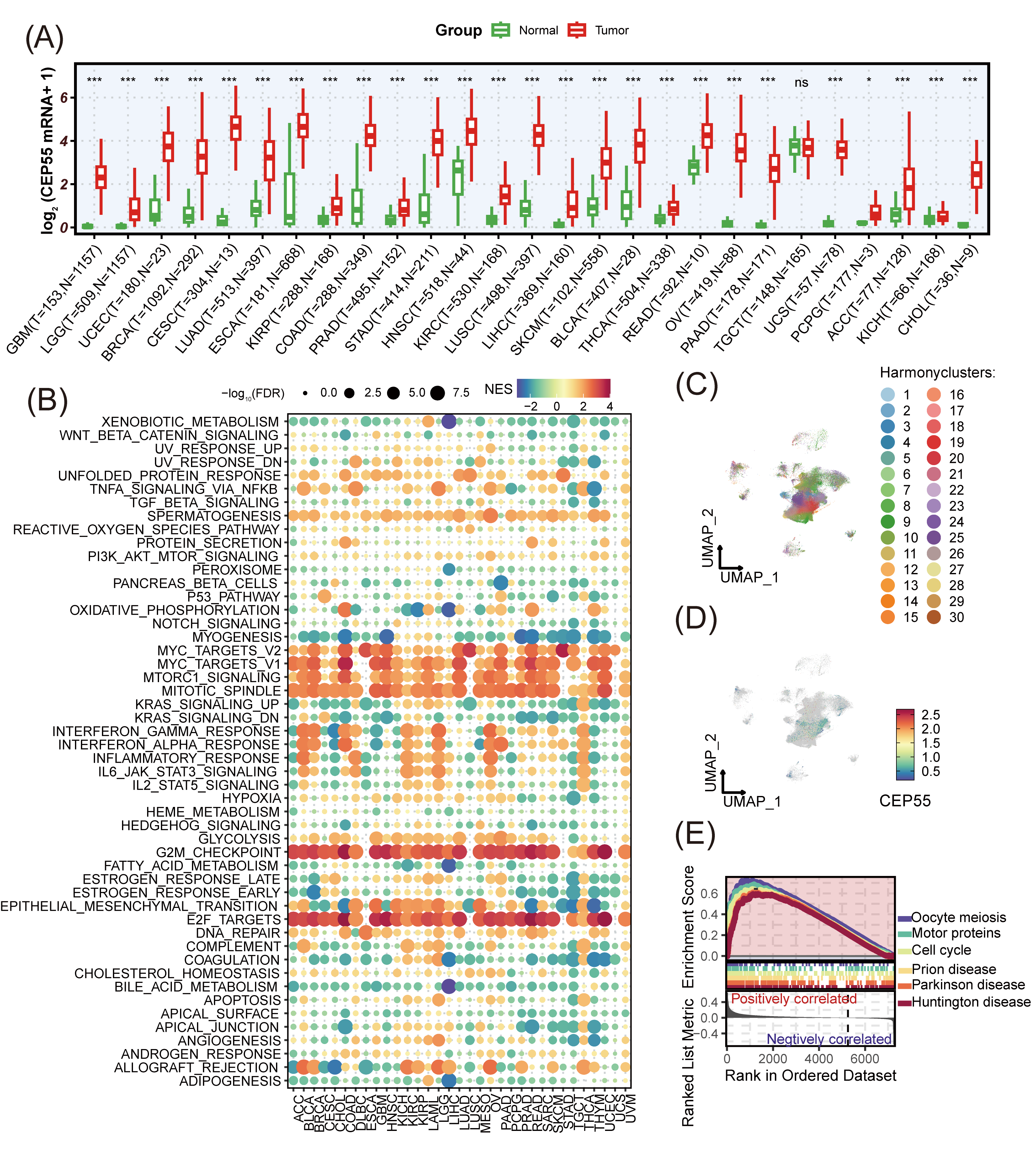


**Figure S10.** Functional Characterization of CEP55 Knockdown Effects. **A.** Western blot confirmation of CEP55 knockout in CT26 and 3LL cells. **B-E.** Quantitative analysis of colony formation (B), invasion (C), and migration (D) assays in CEP55-knockdown CT26 and 3LL cells (n=3 independent experiments). **F-G.** Analysis of T cell exhaustion markers (PD-1 and TIM-3) on CD8+ T cells following co-culture with CEP55-knockdown tumor cells. NS, no significant; **p* < 0.05; ***p* < 0.01; ****p* < 0.001, *****p* < 0.0001.


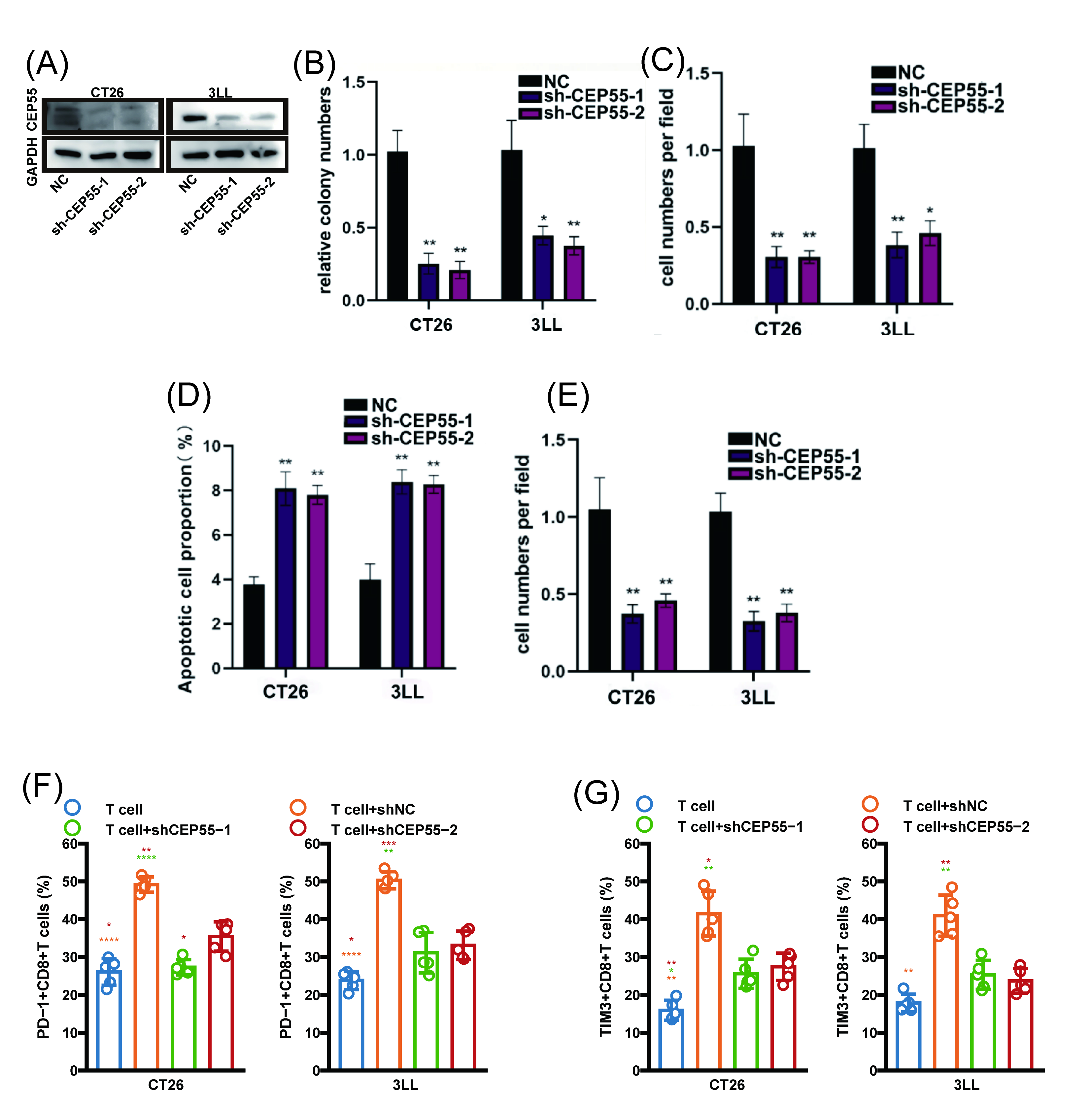

Supplement: Supplementary file 1 — Figure S1: Survival Analysis and Predictive Performance. Figure S2: Distribution and Predictive Performance of integrated Machine Learning and Genetic Algorithm‐driven Multiomics analysis (iMLGAM) Score for immune checkpoint blockade (ICB) Therapy Response Across Multiple Cancer Types. Figure S3: Assessment of iMLGAM score in Multiple Independent Immunotherapy Cohorts. Figure S4: Pan‐Cancer Analysis of iMLGAM Score. Figure S5: Comparative Analysis of Immune Signatures, Cytolytic Activity, and Tumor Microenvironment Features Between iMLGAM Score Groups. Figure S6: Molecular and Immunological Landscape Analysis. Figure S7: Copy Number Alterations in High and Low iMLGAM Score Groups. Figure S8: Quantitative analysis of immune cell populations. Figure S9: Pan‐Cancer Analysis of Centrosomal Protein 55 (CEP55) Expression and Its Associated Molecular Signatures. Figure S10: Functional Characterization of CEP55 Knockdown Effects. [file IMT2-4-e70011-s002.doc]
